# Light-Assisted Akamptisomerization: Excited-State Bond-Angle Reflection (ESBAR) as a Molecular Photoswitching Element in B_2_OF_2_–Porphyrins

**DOI:** 10.1021/acs.inorgchem.6c00108

**Published:** 2026-03-26

**Authors:** Karine N. de Andrade, Jhonathan Rosa de Souza, Paula Homem-de-Mello, Rodolfo G. Fiorot

**Affiliations:** † Department of Organic Chemistry, Institute of Chemistry, 28110 Universidade Federal Fluminense, Outeiro de São João Batista, Niterói, Rio de Janiero 24020-141, Brazil; ‡ Center for Natural and Human Sciences, Federal University of ABC, São Paulo 09280-560, Brazil; § Institut de Química Teòrica i Computacional, 16724 University of Barcelona, Barcelona 08028, Spain

## Abstract

Akamptisomerism arises from bond-angle reflection (BAR) at a B–O–B bridge in low-symmetry B_2_OF_2_ porphyrins and leads to isolable diastereisomers in the ground state. Herein, we present the first theoretical investigation of the excited-state bond-angle reflection (ESBAR). TD-DFT calculations show that the BAR activation barrier decreases from 21.3 kcal mol^–1^ (0.924 eV) in the ground-state (S_0_) to 15.3 kcal mol^–1^ (0.663 eV) in the triplet excited-state (T_1_), rendering the process fluxional upon excitation. The feasibility of singlet–triplet intersystem crossing is supported by near-isoenergetic singlet and triplet states and enhanced spin–orbit coupling. These results provide substantial evidence that akamptisomerism could be facilitated in the excited state, highlighting ESBAR as a potential photoswitching mechanism in porphyrinoid systems.

## Graphical abstract

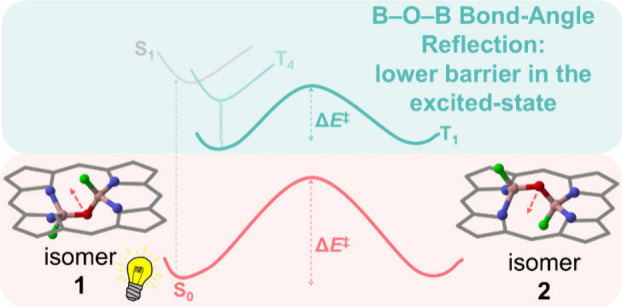

Photoswitches are molecular systems that respond to light as an external stimulus, promoting interconversion between active/inactive states across different regions of the electromagnetic spectrum.^1–3^ This typically involves structural rearrangement of a photoswitching element,^2,4^ most commonly E/Z isomerization around a double bond or ring-closing/opening reactions.^2,5,6^

A typical photoisomerization pathway **A**→**B** involves: (i) photoexcitation of **A**; (ii) vibrational relaxation of the excited specie (A*); (iii) decay through a conical intersection between the excited and ground states; and (iv) barrierless internal conversion to **B** (Figure 1a).^7^ A representative example occurs in human vision where light-induced rotation along the retinal chain in rhodopsin triggers ultrafast photoisomerization and visual signaling. This process has been investigated using surface hopping simulations,^8^ ultrafast spectrocopy,^9^ and more recently QM/MM approaches,^10^ providing realistic descriptions of excited-state dynamics within the protein environment.^10^

**Figure 1:**
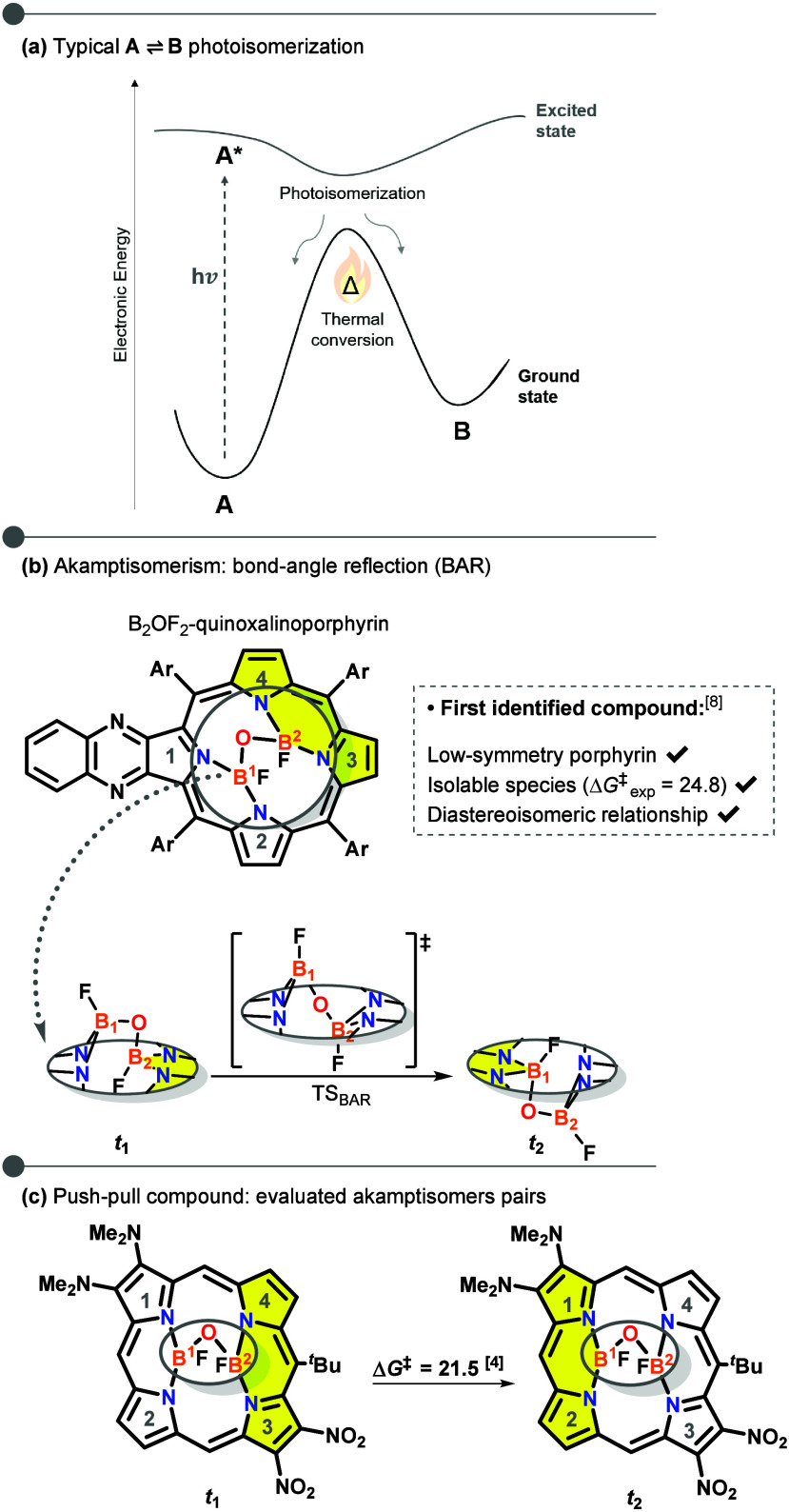
(a) General photoisomerization pathway for the A→B system in the ground and excited states. Akamptisomerism: (b) cut-out view of the B_2_OF_2_-porphyrin core showing bond-angle reflection (BAR) at the B–O–B bridge; (c) this work: most promising compound exhibiting spectral differentiation (pseudoplanes highlighted in yellow). Energies (kcal mol^–1^) were computed at the B3LYP-D3/def2-QZVP//B3LYP-D3/6–31+G** level.

In addition to pathways involving conical intersections, some reactions proceed entirely in excited states, forming a photoisomer that subsequently relaxes to the ground state. This is often observed in excited-state intramolecular proton transfer (ESIPT), which typically involves an enol (**A**)⇌keto­(**B**)^11,12^ isomerization. Upon photoexcitation of the enol form, intramolecular proton transfer generates the keto species, from which fluorescence is emitted.

Photoswitch development has traditionally focused on structural modifications around the switching element.^6^ To expand this field, attention may instead be directed to the switching element itself—the isomerization process. Akamptisomerism, the most recently identified form of conformational isomerism, represents a promising alternative. It arises from bond-angle reflection (BAR) at a B–O–B bridge anchored to low-symmetry porphyrins, first observed in B_2_OF_2_-quinoxalinoporphyrins—Figure 1b.^13,14^ BAR distorts the porphyrin pseudoplanes, yielding diastereoisomers, with distinct physicochemical properties.^13^ Despite this potential, studies remain scarce, mainly limited to Y atom positional preferences in B_2_OY_2_ bridges and associated *transoid*/*cisoid* divergences.^13,15–19^

To the best of our knowledge, only our research group has systematically investigated akamptisomerism by means of theoretical approaches.^4,20^ Fragmentation analysis of B_2_OF_2_-porphyrins revealed that akamptisomerization originates from bridge–macrocycle orbital interactions, enabling BAR with an isolable activation barrier.^20^ A study of 28 akamptisomeric pairs showed that the *transoid* (***t***) BAR remains thermally accessible in the ground state (***t***_**1**_ → ***t***_**2**_, Δ*G* ^⧧^ ≈ 27 kcal mol^–1^), with substituents exerting a minor influence on the energetics.^4^ The average computed activation energies are consistent with the experimentally observed barrier for ***t***_**1**_ → ***t***_**2**_ isomerization in B_2_OF_2_-quinoxalinoporphyrin (∼25 kcal mol^–1^).^13^

Analysis of the absorption profiles identified a minimal β,β push–pull design (−NMe_2_/–NO_2_ on opposite pseudoplanes with *meso*-^*t*^Bu, Figure 1c) that produces pronounced spectral differentiation.^4^ This arrangement yields an energy splitting of 321 meV (7.4 kcal mol^–1^, Δλ = 54 nm) between the ***t***_**1**_ and ***t***_**2**_ in the S_3_ state, attributed to intramolecular charge transfer and steric distortion from the −^*t*^Bu group.^4^ However, the light-induced BAR process itself has not been addressed.

Herein, we report the first DFT-based evidence of excited-state bond-angle reflection (ESBAR) in the akamptisomerism context, demonstrating its feasibility in both singlet and triplet manifolds. In the triplet state, the BAR becomes fluxional (i.e., rapid, nonisolable interconversion),^13^ revealing a potential photoswitching mechanism in porphyrin-based architectures. Ground-state minima, transition states, and IRC pathways were optimized at the B3LYP-D3/6–31+G** level.^21–25^ Excited-state properties were investigated using TD-DFT at the CAM-B3LYP/6–31+G** level.^26^ Selected S_1_ and T_1_ structures along the BAR pathway were optimized with constrained B–O–B angles, and spin–orbit coupling (SOC) matrix elements were computed to assess intersystem crossing (ISC).^27^ Full computational details are provided in the Supporting Information (SI).

To investigate the light-assisted BAR processes, we examined the excited-state potential energy surfaces of the promising compound—Figure 1c. The optimized ground state (S_0_) geometries of the akamptisomers ***t***_**1**_ and ***t***_**2**_, along with the corresponding B–O–B bond-angle reflection transition state (TS), and their B–O–B bond angles are shown in Figure S2 in the SI. This TS is characterized by a near-linear B–O–B bridge (B–O–B bond angle: 174.9°), consistent with a BAR pathway. In addition to the singlet manifold, ESBAR was investigated on the T_1_ surface, motivated by the potential involvement of ISC. This was evaluated through SOC matrix elements (Ĥ_SO_), whose squared magnitude, |Ĥ_SO_|^2^, provides a qualitative measure of the coupling efficiency between states of different spin multiplicities.

Spin–orbit coupling (SOC) matrix elements were computed between singlet (S_*m*_, *m* = 0–2) and triplet (T_*n*_, *n* = 1–4) states at the optimized akamptisomer minima (***t***_**1**_ and ***t***_**2**_). Within the singlet manifold, S_0_ and S_1_ were the primary focus, while S_2_ was included to provide a broader view of the low-lying excited states. Triplet states were selected based on energetic proximity to the singlets; thus, T_1_–T_4_ were examined, with T_4_ lying closest in energy to S_1_. Table 1 summarizes the squared SOC matrix elements |Ĥ_SO_|^2^ and the corresponding singlet–triplet energy gaps, enabling evaluation of ISC feasibility between S_0_–S_2_ (rows) and T_1_–T_4_ (columns). The table reports both the squared SOC matrix elements |SO|^2^ and associated energy differences. Notably, three triplet states lie between S_0_ and S_1_ with small energy gaps, while T_4_ is nearly isoenergetic with S_1_ (Δ*E*_S‑T_ ≈ 10^–2^ eV) for both akamptisomers.

**Table 1:** Squared Spin-Orbit Coupling Matrix Elements, |*Ĥ*_SO_|^2^, and Singlet-Triplet Energy Gap (Δ*E*_S‑T_, eV) for the Lowest-Lying Singlet and Triplet States of the Akamptisomer Pair (***t***_**1**_ and ***t***_**2**_), Computed at the TD-CAM-B3LYP/6-31+G** Level

		|*Ĥ*_SO_|^2^ (cm^–1^) (Δ*E*_S‑T_ (eV))			
		T_1_	T_2_	T_3_	T_4_
***t***_**1**_	S_0_	28.315 *(1.01)*	29.274 *(1.60)*	25.399 *(1.61)*	51.225 *(1.82)*
	S_1_	7.108 (0.64)	10.215 (0.22)	15.445 (0.14)	17.665 (*−0.02*)
	S_2_	27.286 (1.05)	10.937 (0.62)	22.314 (0.55)	8.586 (0.38)
					
***t***_**2**_	S_0_	27.286 *(0.89)*	59.998 *(1.64)*	32.948 *(1.76)*	43.286 *(2.30)*
	S_1_	18.560 (0.70)	7.257 (0.29)	44.275 (0.14)	57.270 (0.01)
	S_2_	64.030 (1.08)	12.538 (0.67)	49.416 (0.76)	33.754 (0.37)

First, considering the singlet–triplet energy gaps (values in parentheses), the small separations qualitatively favor ISC, since reduced energy differences generally enhance the probability of spin-state crossing. Accordingly, an ISC process from S_1_ to T_4_ in the akamptisomer ***t***_1_ is plausible, as this transition exhibits the smallest computed gap among the evaluated states (Δ*E*_S_1_‑T_4__ = −0.02 eV). For comparison, some organic compounds with experimental evidence of triplet-state population show computed Δ*E*_S‑T_ = 0.27–0.48 eV (TD-B3LYP/6–31G).^28^ Nevertheless, ISC from S_1_ to T_3_ and T_2_ is also possible given that the energy gaps between these states are small. However, only the T_4_ state is discussed in this work.

Regarding SOC, the computed values are larger for S_1_→T_4_ than for S_1_→T_1_ (akamptisomers ***t***_**1**_: 17.665 vs 7.108 cm^–1^; ***t***_**2**_: 57.270 vs 18.560 cm^–1^), indicating that ISC via S_1_→T_4_ is favored due to stronger SOC and near-isoenergetic states. Notably, SOC values above 1.0 cm^–1^ could be sufficient to promote ISC.^29^ Comparable SOC magnitudes (10–30 cm^–1^) have been reported for S_1_→T_*n*_ transitions in benzophenone-containing difluoroboron β-diketonates, correlating with efficient ISC and observed phosphorescence profiles.^30^ In B_2_OF_2_-porphyrinoids, bridge anchoring is known to alter the emission profile and quenches fluorescence, suggesting deactivation through nonradiative decay or triplet state population.^31,32^ Population of the triplet manifold is particularly relevant because the increased excited-state lifetime enables photosensitized processes under low-energy conditions.^33^

TheoDORE^34^ analysis shows that S_1_ and T_4_ of akamptisomer ***t***_**1**_ possess similar localized excitation (LE) and charge-transfer (CT) contributions, indicating mixed LE/CT character. Molecular orbital inspection reveals that the dominant configurations (HOMO→LUMO for S_1_; HOMO→LUMO+1 for T_4_) differ in spatial distributions (see Figure S4 in the SI), favoring ISC between states of distinct orbital character.^35^ Excited-state nature evaluation is provided in Section S3 in the SI. Consistent with the small singlet–triplet gap, computed spin–orbit coupling values, and orbital analysis, the S_1_→T_4_ ISC is expected to be efficient, following by a rapid internal conversion to the lowest triplet state (T_1_).

Considering the potential triplet-state population, and in accordance with Kasha’s rule^36^—which states that emission occurs predominantly from the lowest excited state of a given multiplicity—the ESBAR process was evaluated for both S_1_ (at TD-DFT level) and T_1_ (at DFT level). Nine points were computed along the IRC, equally spaced according to the steps generated during the IRC calculation. For both singlet (S_1_) and triplet (T_1_) states, full geometry optimizations were performed for the akamptisomers ***t***_**1**_ and ***t***_**2**_; their optimized structures and corresponding B–O–B bond angles are shown in Figure S3 in the SI). Constrained optimizations were carried out at the transition state, and intermediate geometries were obtained from the IRC calculations by fixing B–O–B bond angle. The resulting energy diagram (Figure 2) depicts the overall ***t***_**1**_ → ***t***_**2**_ isomerization pathway in the ground (S_0_) and excited (S_1_ and T_1_) states.

**Figure 2:**
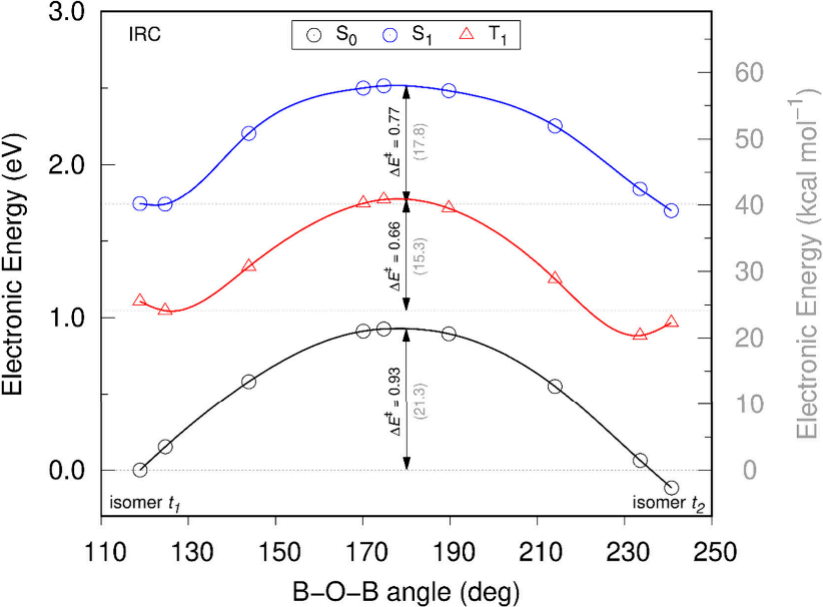
IRC energy profiles for B–O–B bond-angle reflection along the ***t***_**1**_ → ***t***_**2**_ isomerization pathway. Curves correspond to the ground (S_0_) and excited (S_1_ and T_1_) states. Evaluated geometries span B–O–B bond angles of 118.9° (***t***_**1**_), 124.7°, 143.9°, 170.2°, 174.9° (TS_BAR_), 189.7°, 214.1°, 233.6°, and 240.8° (***t***_**2**_). Energies were computed at the CAM-B3LYP/6–31+G** level and are reported in kcal mol^–1^ (left axis, bold font, black) and eV (right axis, italic font, gray).

The computed energy diagram reveals an unconventional profile compared to classical photoswitches, which typically involve conical intersections between ground and excited states (Figure 1a). Photoexcitation significantly reduces the isomerization barriers, with activation energies of Δ*E* ^⧧^ = 17.8 and 15.3 kcal mol^–1^ for the S_1_ and T_1_ states, respectively, relative to the corresponding excited-state minimum of ***t***_**1**_. In T_1_, the barrier is reduced by 6.0 kcal mol^–1^, compared to that of the S_0_ BAR pathway.

Because this unimolecular ESBAR barrier lies below 16 kcal mol^–1^, the triplet-state pathway corresponds to a low-energy conformational rearrangement with an estimated first-order half-life of ∼1 ms at 298 K, rendering the akamptisomers nonisolable.^13,37,38^ Akamptisomerism thus becomes fluxional^13,39^ in the excited triplet state, effectively operating as a photoisomerization process.

To provide an overall view of the photophysical behavior, we evaluated the de-excitation pathways of both akamptisomers (***t***_**1**_ and ***t***_**2**_) and constructed the Jablonski diagram (Figure 3). Details of the fluorescence and phosphorescence calculations are provided in Table S1 in the SI. Both akamptisomers display large Stokes shifts, with near-infrared emission (λ > 700 nm). Accordingly, the reduced triplet-state BAR barrier, considerable SOC between near-isoenergetic S_1_ and T_4_ states, and computed emission profiles collectively support the proposed excited-state pathways summarized in Figure 3. Although explicit excited-state rate constants and vibrational couplings were not computed, as they are beyond the scope of this communication, these results suggest that ESBAR may kinetically compete with radiative and nonradiative relaxation pathways, particularly within a long-lived triplet manifold.

**Figure 3:**
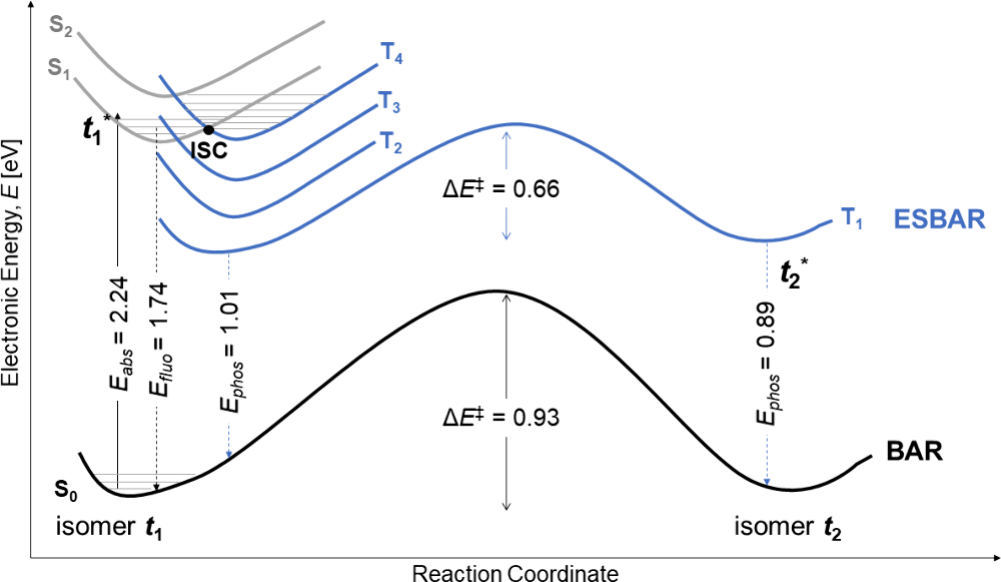
Jablonski-like diagram depicting the ESBAR process for the ***t***_**1**_→ ***t***_**2**_ isomerization in the ground (S_0_, black curve) and excited states (S_1_, gray; T_1_, blue). Excitation of ***t***_**1**_ (absorption, *E*_abs_) is followed by possible deactivation pathways: fluorescence (*E*_fluo_), intersystem crossing (ISC) to T_4_, phosphorescence (*E*_phos_) from T_1_, and/or akamptisomerization to ***t***_**2**_ via ESBAR in T_1_. Energies are given in eV, and curves were constructed from IRC-derived points.

In this scheme, excitation of akamptisomer ***t***_**1**_ to S_1_ is followed by a likely intersystem crossing to T_4_, and rapidly relaxation to the lowest-triplet T_1_. The BAR is favored on the T_1_ surface, where the activation barrier is lower than those in both S_1_ (Δ*E* ^⧧^ = 0.77 eV) and S_0_ (Δ*E* ^⧧^ = 0.93 eV). The clear involvement of ISC and triplet population underscores the photoactive potential of these systems.

We report the first DFT/TD-DFT investigation of excited-state bond-angle reflection (ESBAR) in B_2_OF_2_ porphyrins. Exploration of the S_1_ and T_1_ manifolds reveals that ESBAR is facilitated by singlet–triplet ISC, supported by the near-isoenergetic S_1_/T_4_ states (Δ*E* ≈ 10^–2^ eV) and sizable spin–orbit coupling values. The BAR barrier decreases from 21.3 kcal mol^–1^ in S_0_ to 15.3 kcal mol^–1^ in the T_1_ surface, rendering akamptisomerization fluxional in the excited state. These findings provide the first theoretical evidence that BAR can be light-assisted, establishing ESBAR as a potential photoswitching mechanism whose practical utility will depend on the nature of the functional units attached to the porphyrinoid framework. Given the recent emergence of akamptisomerism and the scarcity of experimental reports, we anticipate that these findings will motivate experimental fluorescence and phosphorescence studies of B_2_OF_2_ porphyrins to probe the involvement of low-lying triplet states and near-infrared emission.

## Supplementary Material



## Notes

Details regarding computational methodology, additional electronic and geometric information, as well as all Cartesian coordinates (PDF)
