# Supplementary material for: Light-Assisted Akamptisomerization: Excited-State Bond-Angle Reflection (ESBAR) as a Molecular Photoswitching Element in B2OF2–Porphyrins
Source: Inorg Chem. 2026 Mar 26;65(13):7093–7. doi: 10.1021/acs.inorgchem.6c00108 (PMC13058890; doi:10.1021/acs.inorgchem.6c00108)
Supplement: Supplementary file 1 [file ic6c00108_si_001.pdf]

SUPPORTING INFORMATION

Light-Assisted Akamptisomerization: Excited-State Bond-Angle Reflection (ESBAR) as a Molecular Photoswitching Element in B_2OF_2-Porphyrins

Karine N. de Andrade,[1] Jhonathan Rosa de Souza,[2,3] Paula Homem-de-Mello,[3] Rodolfo G. Fiorot[1,*]

[1] Department of Organic Chemistry, Institute of Chemistry, Universidade Federal Fluminense, Outeiro de São João Batista, Niterói, Rio de Janiero 24020-141, Brazil. [2] Center for Natural and Human Sciences, Federal University of ABC, São Paulo, Brazil [3] Institut de Química Teòrica i Computacional, University of Barcelona, Barcelona 08028, Spain.

*Corresponding author: rodolfofiorot@id.uff.br

TABLE OF CONTENTS

1. **Computational methodology** S2
2. **Akamptisomers: optimized structures** S3
3. **Nature of excited states (singlet and triplet) — akamptisomers t_1** S4
4. **De-excitation processes** S6
5. **Coordinate matrices** S6
6. **References** S23

1. Computational methodology

Density Functional Theory (DFT) calculations were employed throughout this work. Ground state optimizations of akamptisomers, transition states, and intrinsic reaction coordinate (IRC) pathways were carried out using *Gaussian 09*[1], using the B3LYP[2,3] functional combined with the 6-31+G**[4,5] basis set. Long-range dispersion interactions were accounted for by Grimme's D3[6] empirical correction. The B3LYP-D3/6-31+G** level was selected based on our previous study of akamptisomerism in porphyrinoid systems[7,8], as well as the seminal report of Canfield and co-workers [9] on BAR and related processes. For B_2OF_2-porphyrins, we have shown that both the BAR activation energy and key geometric parameters are weakly dependent on the functional choice, with comparable results obtained using B3LYP-D3, M06-2X and CAM-B3LYP, associated with def2-TZVP basis set.[8] These results support the reliability of the chosen level for mapping ground state reaction pathways. The nature of all stationary points was confirmed by vibrational frequency analyses: minima (akamptisomers) showed no imaginary frequencies, while transition states presented exactly one imaginary frequency. IRC calculations were carried out to identify the minimum-energy pathways connecting akamptisomers.

Excited-state properties, including the energy reported to elaborate the energy profile in the excited-states, were simulated using time-dependent DFT (TD-DFT) at the CAM-B3LYP[10]/6-31+G** level. This functional was chosen due to its proven performance in describing charge-transfer excitations and absorption spectra of porphyrin-like systems, including B_2OF_2-porphyrins.[11–14] Excited-state potential energy profiles were explored through vertical excitation of geometries obtained along the ground-state IRC and constrained scans, an approach widely adopted in TD-DFT studies of excited-state reactions.[7,15–17] To investigate excited-state bond angle reflection (ESBAR), points along the IRC pathway, transition state, and akamptisomers were optimized in the first singlet (S_1) and triplet (T_1) states, with the B–O–B bond angle constrained, at the TD-B3LYP-D3/6-31+G** level. Single-point calculations were subsequently performed at the TD-CAM-B3LYP/6-31+G** level (*nstates* = 20, singlets) to map the B–O–B BAR profile. Spin–orbit coupling (SOC) constants for singlet-triplet transitions were obtained at the TD-DFT level using quasi-degenerate perturbation theory [18], to probe intersystem crossing (ISC) and the triplet-state contribution to ESBAR. To do so, the

elements of spin-orbit coupling operator matrix, $\hat{H}_{SO}$, were computed. All ESBAR calculations were performed with *ORCA 5.0.3*.[19,20] The nature of the singlet and triplet excited states involved in the potential intersystem crossing, specifically S_1 and T_4, was assigned using a fragment-based analysis of the transition density matrix performed with the TheoDORE 3.1.1 package[21]. Figure S1a presents the overall workflow for the excited-state analysis, while Figure S1b shows the molecular fragmentation scheme employed in the TheoDORE calculations.

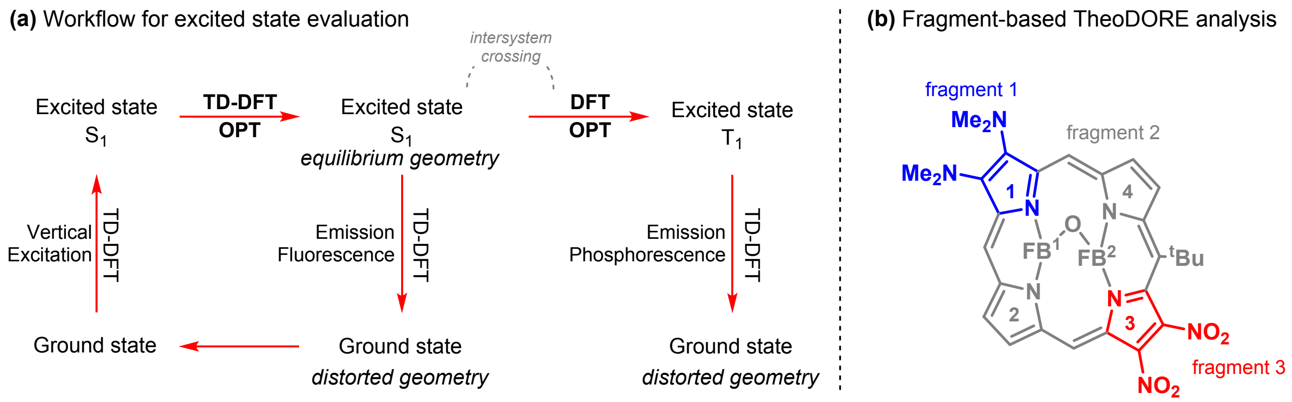

Figure S1: (**a**) Schematic representation of the excitation and de-excitation processes explored. (**b**) Fragmentation scheme employed in the TheoDORE analysis of the transition density matrix. The electron-donating fragment is shown in blue (pyrrolic ring 1 plus donating substituent), the porphyrin core connector in gray, and the electron-withdrawing fragment in red (pyrrolic ring 3 plus accepting substituent)

2. Akamptisomers: optimized structures

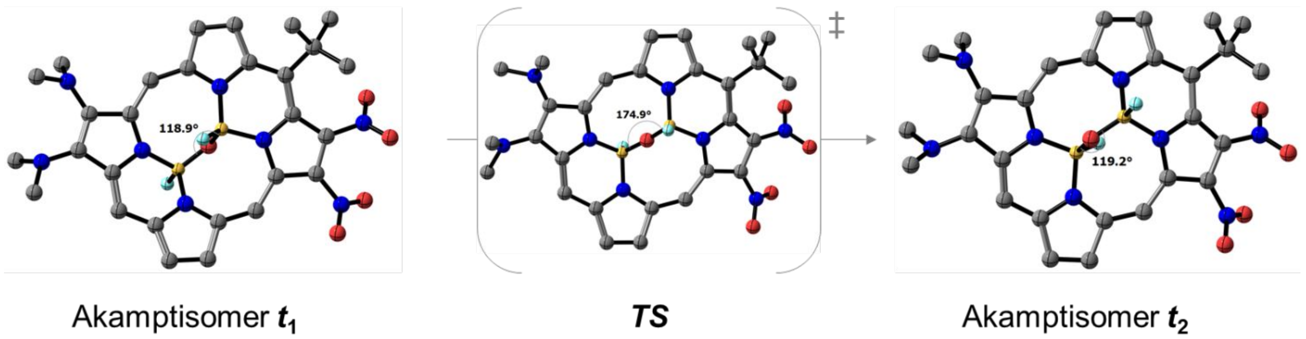

Figure S2: Optimized structures of akamptisomers t_1 and t_2, and the nearly linear transition state (***TS***), obtained at the B3LYP-D3/6-31+G** level. Hydrogen atoms were omitted for clarity.

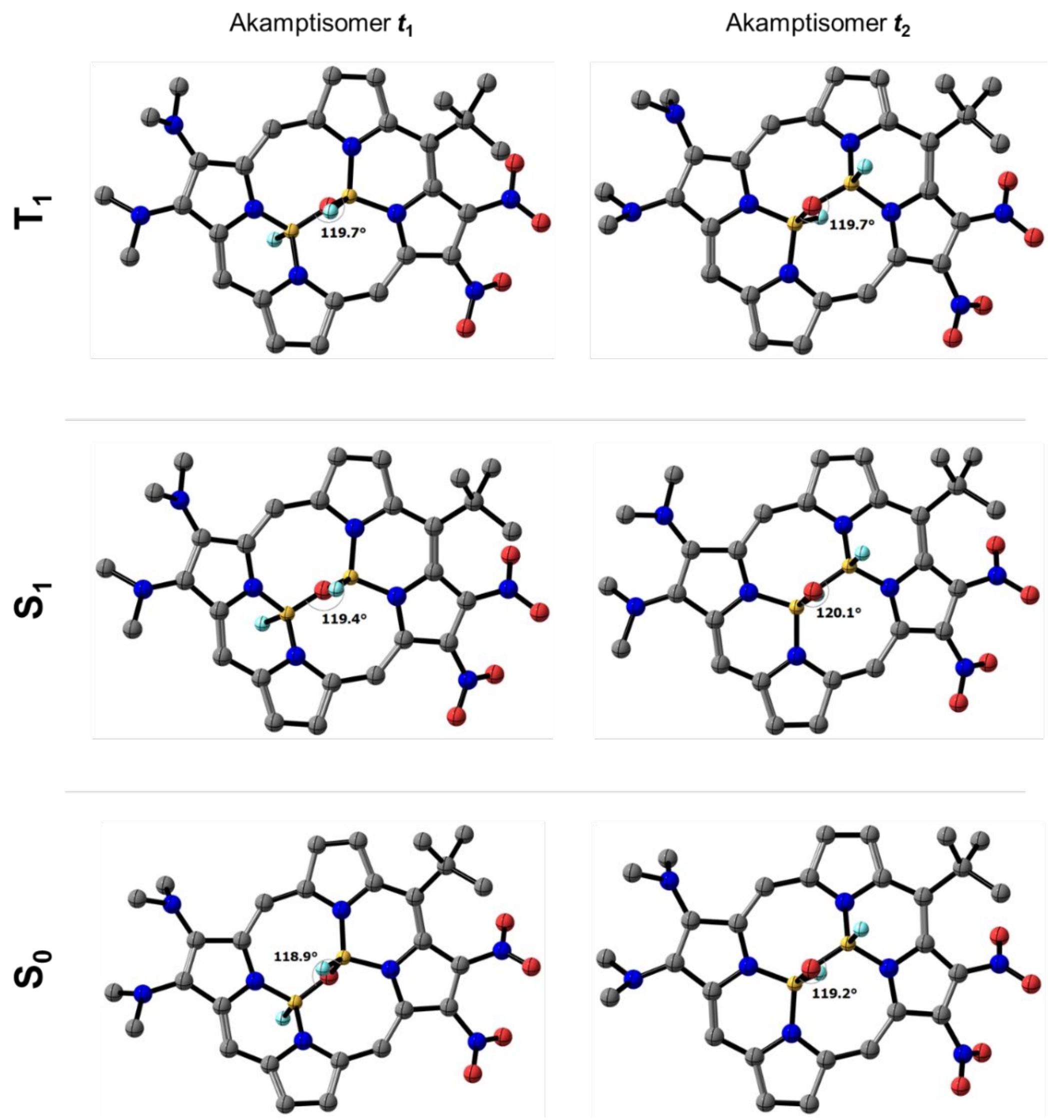

Figure S3: Optimized structures of akamptisomers $\boldsymbol{t_1}$ and $\boldsymbol{t_2}$ in the ground state (S_0) and in the first excited singlet (S_1) and triplet (T_1) states. All structures correspond to fully optimized minima at the B3LYP-D3/6-31+G** level. Hydrogen atoms were omitted for clarity.

3. Nature of excited states (singlet and triplet) — akamptisomers $\boldsymbol{t_1}$

The nature of the excited states relevant to ISC (S_1 and T_4) in akamptisomer $\boldsymbol{t_1}$ was analyzed using transition density matrix calculations based on the fragmentation scheme shown in Figure S1b, together with inspection of the dominant molecular orbitals involved in the electronic transitions. The excitation character was quantified in terms of localized excitation (LE) and charge-transfer (CT) contributions, with transition densities normalized such that the total Ω equals unity for all states. The results indicate 53% CT and 47% LE contributions for both states, suggesting partially overlapping charge distributions and a mixed LE/CT character with nearly identical compositions.

Regarding the molecular orbital analysis, the dominant configurations correspond to HOMO → LUMO for S_1 and HOMO → LUMO+1 for T_4 (Figure S4). A qualitative inspection of the orbitals shows that the HOMO is mainly localized over the donor fragment and the directly attached pyrrolic ring, whereas both LUMO and LUMO+1 are predominantly distributed over the pyrrolic rings and the acceptor fragment, consistent with a mixed LE/CT profile and in agreement with the TheoDORE fragmentation analysis.

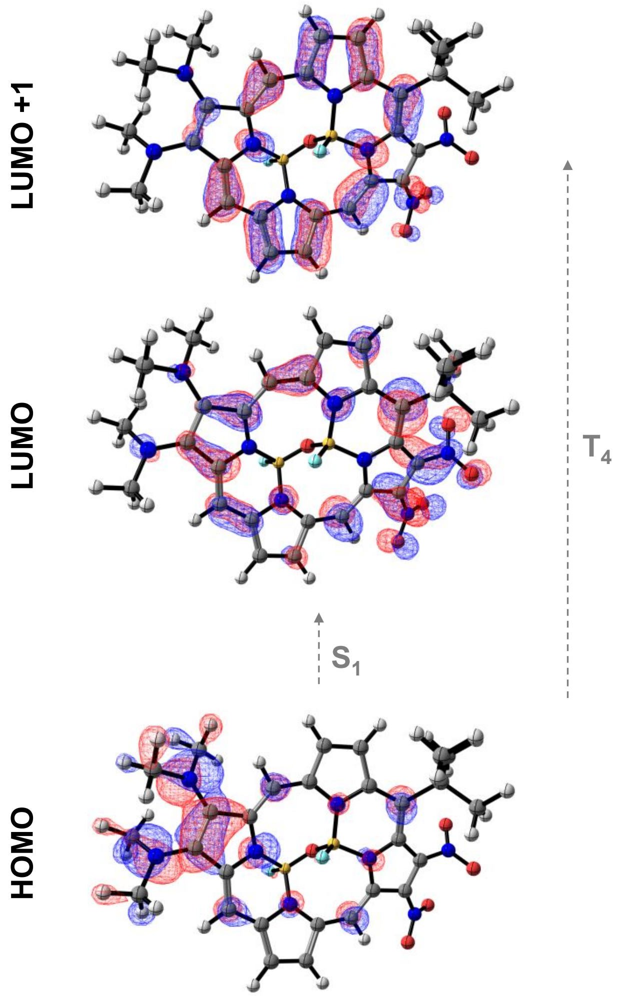

Figure S4: Frontier molecular orbitals associated with the main electronic configurations contributing to the S_1 (HOMO → LUMO) and T_4 (HOMO → LUMO+1) excited states, computed at the CAM-B3LYP/6-31+G** level of theory.

Importantly, LUMO and LUMO+1 exhibit distinct spatial distributions, with lobes oriented nearly perpendicular to each other, noticeable phase changes, and different delocalization planes. This variation in orbital character between S_1 and T_4 is consistent with El-Sayed's rule[22], which states that intersystem crossing is favored when the involved states differ in orbital type or spatial character. Therefore, despite the similar LE/CT contributions from the selected fragments, the spatial reorganization of the acceptor orbital supports the feasibility of the S_1 → T_4 intersystem crossing.

4. De-excitation processes

The de-excitation processes of the akamptisomers were investigated through their fluorescence ($S_1 \rightarrow S_0$) and phosphorescence ($T_1 \rightarrow S_0$) pathways. Fluorescence was computed from the equilibrium geometry of the S_1 state, according to Equation (1):

$$\Delta E_{\mathrm{fluo}} = ES_1\ (R_{1'}) - ES_0(R_{1'}) \qquad (1)$$

where $R_{1'}$ represents the relaxed geometry at the excited singlet state (S_1). Phosphorescence was evaluated from the triplet population reached through intersystem crossing (ISC), as described by Equation (2):

$$\Delta E_{\mathrm{phos}} = ET_1\ (R_{3'}) - ES_0(R_{3'}) \qquad (2)$$

where $R_{3'}$ corresponds to the relaxed geometry at the triplet state (T_1). Both processes were treated as vertical emissions, following the Franck-Condon principle, which assumes that the electronic transition occurs much faster than nuclear reorganization. Table S1 summarizes the computed emission energies (eV) and wavelengths (nm), as well as the corresponding Stokes shifts for the fluorescence processes.

Table S1: Fluorescence and phosphorescence energies and their corresponding emission wavelength. Stokes shifts were computed as $|\Delta E| = |E_{\mathrm{emission}}(S_1 \rightarrow S_0) - E_{\mathrm{absorption}}\ (S_0 \rightarrow S_1)|$ and $\Delta\lambda = \lambda_{\mathrm{emission}}(S_1 \rightarrow S_0) - \lambda_{\mathrm{absorption}}\ (S_0 \rightarrow S_1)$. Computed values at the CAM-B3LYP/6-31+G** level.

Akamptisomers	Fluorescence		Stokes shift		Phosphorescence	
	E (eV)	λ (nm)	$\|\Delta E\|$ (eV)	Δλ (nm)	ΔE (eV)	λ (nm)
$\boldsymbol{t_1}$	1.74	710	0.50	158	1.01	1229
$\boldsymbol{t_2}$	1.81	684	0.47	140	0.89	1393

From the computed data, a pronounced Stokes shift was identified for the fluorescence process (Table S1, $|\Delta E|$ and Δλ), with emission occurring in the near-infrared (NIR) region (λ > 700 nm). In contrast, the phosphorescence transition is predicted to lie well beyond the visible range, rendering it experimentally inaccessible under conventional conditions. Similar photophysical behavior has been reported for modified porphyrinoid systems exhibiting low-energy NIR emission.[23–25]

5. Coordinate matrices

1. Akamptisomer $\boldsymbol{t_1}$, BÔB = 118.9°

Ground-state geometry, S_0:

F	-0.228553000	0.122808000	-1.305179000
B	-1.347160000	1.139621000	0.963476000
B	0.392923000	-0.151255000	-0.062377000
O	-0.191811000	0.399635000	1.091269000
F	-1.851397000	1.621118000	2.185560000
C	-0.504744000	4.394621000	-0.785243000
C	-0.035007000	3.129696000	-0.234244000
C	1.332192000	2.849004000	-0.133349000
C	2.160091000	1.700072000	-0.027361000
C	3.581790000	1.746648000	0.137856000
C	4.068722000	0.452153000	0.131108000
C	2.967766000	-0.438493000	-0.099401000
C	2.860633000	-1.862911000	-0.275550000
C	1.595927000	-2.432699000	-0.007133000
C	1.195389000	-3.806710000	0.229716000
C	-0.143165000	-3.807060000	0.497187000
C	-0.622791000	-2.443216000	0.418477000

C	-1.995861000	-2.124917000	0.531536000
C	-2.821236000	-0.998954000	0.402665000
C	-4.268515000	-1.140258000	0.176973000
C	-4.754128000	0.105373000	-0.207810000
C	-3.624020000	1.022170000	-0.146285000
C	-3.512062000	2.384943000	-0.450381000
C	-2.260991000	3.026691000	-0.430324000
C	-1.862635000	4.323263000	-0.923790000
N	-1.153261000	2.368337000	0.009758000
N	1.852738000	0.365790000	-0.130718000
N	0.462213000	-1.665432000	0.134788000
N	-2.515420000	0.329337000	0.271355000
H	1.847668000	-4.664093000	0.246591000
H	-0.761718000	-4.660743000	0.739709000
H	1.930445000	3.742739000	-0.273201000
H	-2.590226000	-3.029244000	0.622781000
H	-4.369676000	2.942129000	-0.796499000
C	3.982638000	-2.763334000	-0.866591000
C	5.134917000	-1.976532000	-1.542875000
C	4.598558000	-3.691142000	0.208391000
C	3.365609000	-3.607486000	-2.022895000
H	4.775015000	-1.108979000	-2.103522000
H	5.906006000	-1.662341000	-0.845199000
H	5.632013000	-2.638745000	-2.256871000
H	3.863522000	-4.369616000	0.646315000
H	5.387670000	-4.299517000	-0.246983000
H	5.040318000	-3.102073000	1.014767000
H	4.148134000	-4.236763000	-2.458750000
H	2.552930000	-4.260926000	-1.711642000
H	2.980068000	-2.951142000	-2.810540000
N	-4.925957000	-2.368207000	0.216749000
N	-6.082448000	0.389528000	-0.535364000
C	-5.158538000	-2.982663000	1.523620000
H	-5.214583000	-4.071922000	1.416823000
H	-4.341152000	-2.738528000	2.204761000
H	-6.099724000	-2.637566000	1.984124000
C	-5.950599000	-2.654987000	-0.779621000
H	-5.666652000	-2.210968000	-1.734789000
H	-6.020247000	-3.741697000	-0.904446000
H	-6.947448000	-2.277055000	-0.506485000
C	-7.064279000	0.268667000	0.541657000
H	-8.057833000	0.090249000	0.115835000
H	-6.809724000	-0.568999000	1.190608000
H	-7.106444000	1.181626000	1.159592000
C	-6.392654000	1.440282000	-1.491704000
H	-5.635060000	1.466898000	-2.277900000
H	-7.360328000	1.221411000	-1.957374000
H	-6.467876000	2.440211000	-1.030686000
H	-2.526592000	5.062278000	-1.350988000
H	0.148147000	5.202324000	-1.088629000
N	5.425533000	0.113290000	0.496922000
O	5.565040000	-0.759560000	1.355834000
O	6.336373000	0.717381000	-0.063709000
N	4.355601000	2.935114000	0.440219000
O	5.142529000	2.873723000	1.380395000
O	4.158252000	3.930852000	-0.262547000

E = –2146.5186 a. u.

Excited-state geometry, S_1:

F	0.233937000	0.106059000	1.281347000
B	1.335557000	1.188699000	-0.942479000
B	-0.407638000	-0.138678000	0.036789000
O	0.208685000	0.427672000	-1.099409000
F	1.883552000	1.669746000	-2.145119000
C	0.481435000	4.384433000	0.851843000
C	0.021211000	3.119067000	0.297260000
C	-1.345267000	2.843104000	0.193996000
C	-2.184326000	1.709388000	0.042575000
C	-3.611695000	1.768118000	-0.131240000
C	-4.093176000	0.450595000	-0.149946000
C	-2.980719000	-0.429170000	0.066496000
C	-2.859528000	-1.847572000	0.263682000
C	-1.596682000	-2.413590000	-0.031884000
C	-1.199075000	-3.782372000	-0.289124000
C	0.137712000	-3.775911000	-0.583788000
C	0.614616000	-2.415717000	-0.497229000
C	1.979815000	-2.094928000	-0.613456000
C	2.830442000	-0.983776000	-0.457347000
C	4.272439000	-1.120132000	-0.205850000
C	4.768829000	0.166916000	0.163500000
C	3.637503000	1.064248000	0.154318000
C	3.506592000	2.428272000	0.478049000
C	2.258191000	3.049380000	0.476092000
C	1.842864000	4.335860000	0.973392000
N	1.148317000	2.371959000	0.038917000
N	-1.862729000	0.376190000	0.107426000
N	-0.473637000	-1.642677000	-0.186678000
N	2.554211000	0.346474000	-0.284560000
H	-1.851972000	-4.639094000	-0.302571000
H	0.751057000	-4.627559000	-0.846955000
H	-1.946315000	3.731043000	0.350099000
H	2.537395000	-3.008857000	-0.758573000
H	4.360238000	3.003182000	0.801542000
C	-3.945202000	-2.748911000	0.905435000
C	-5.060680000	-1.957253000	1.629610000
C	-4.602251000	-3.694234000	-0.126598000
C	-3.268605000	-3.580506000	2.034870000
H	-4.666281000	-1.095100000	2.175121000
H	-5.853825000	-1.628789000	0.965039000
H	-5.529764000	-2.619525000	2.362979000
H	-3.879112000	-4.369581000	-0.589719000
H	-5.360009000	-4.306546000	0.374985000
H	-5.088992000	-3.117022000	-0.913726000
H	-4.028583000	-4.203416000	2.516882000
H	-2.473513000	-4.236493000	1.684890000
H	-2.842785000	-2.915872000	2.794229000
N	5.016771000	-2.267020000	-0.223866000
N	6.083177000	0.469661000	0.403253000
C	4.812071000	-3.338115000	-1.192048000
H	4.326121000	-4.212671000	-0.741469000
H	4.223581000	-2.979826000	-2.034937000
H	5.794284000	-3.655685000	-1.562987000
C	5.988997000	-2.576179000	0.820607000
H	6.022917000	-1.774015000	1.554907000
H	5.684037000	-3.503988000	1.320811000
H	6.989920000	-2.729021000	0.400635000
C	7.145916000	-0.033865000	-0.465121000
H	7.940851000	-0.505717000	0.122844000
H	6.746522000	-0.752688000	-1.177510000
H	7.582061000	0.805056000	-1.023123000
C	6.469573000	1.620789000	1.207504000
H	5.736190000	1.791759000	1.994505000
H	7.436096000	1.404429000	1.673466000
H	6.580933000	2.529307000	0.600009000
H	2.497654000	5.084983000	1.397343000
H	-0.182700000	5.179929000	1.161299000

N	-5.426932000	0.066429000	-0.471539000
O	-5.567721000	-0.890692000	-1.257868000
O	-6.359000000	0.690054000	0.046813000
N	-4.347898000	2.940302000	-0.468190000
O	-5.303060000	2.823206000	-1.246775000
O	-3.977698000	4.029391000	0.025503000

E = –2146.4682 a. u.

Excited-state geometry, T_1:

F	0.219906000	0.107188000	1.314671000
B	1.335623000	1.183793000	-0.952856000
B	-0.401625000	-0.134689000	0.066356000
O	0.184355000	0.434642000	-1.076987000
F	1.809369000	1.697551000	-2.175691000
C	0.473307000	4.409571000	0.845249000
C	0.009180000	3.142652000	0.285477000
C	-1.351395000	2.860730000	0.195762000
C	-2.198780000	1.707263000	0.038261000
C	-3.600703000	1.749848000	-0.174333000
C	-4.073683000	0.420855000	-0.204843000
C	-2.975269000	-0.432236000	0.057529000
C	-2.839485000	-1.868310000	0.282218000
C	-1.566199000	-2.429643000	-0.043889000
C	-1.171499000	-3.791259000	-0.334597000
C	0.164453000	-3.773867000	-0.629472000
C	0.640067000	-2.415254000	-0.513378000
C	1.992344000	-2.081795000	-0.659344000
C	2.844479000	-0.954178000	-0.499831000
C	4.261459000	-1.099774000	-0.279635000
C	4.750750000	0.182508000	0.158365000
C	3.621049000	1.069243000	0.139213000
C	3.494516000	2.441161000	0.472248000
C	2.240923000	3.061503000	0.470566000
C	1.829758000	4.353006000	0.974167000
N	1.138180000	2.391443000	0.028256000
N	-1.868331000	0.376677000	0.139824000
N	-0.457242000	-1.650115000	-0.169565000
N	2.524560000	0.372628000	-0.298342000
H	-1.825337000	-4.647543000	-0.361763000
H	0.780130000	-4.617915000	-0.911876000
H	-1.955495000	3.743895000	0.369774000
H	2.558710000	-2.980868000	-0.863405000
H	4.345544000	3.015017000	0.803631000
C	-3.891706000	-2.747381000	0.999396000
C	-4.978460000	-1.923800000	1.733287000
C	-4.587781000	-3.728472000	0.023406000
C	-3.167955000	-3.548942000	2.123008000
H	-4.552398000	-1.075169000	2.276815000
H	-5.765830000	-1.566083000	1.075595000
H	-5.465561000	-2.572412000	2.467243000
H	-3.882160000	-4.424215000	-0.436368000
H	-5.327940000	-4.319468000	0.574837000
H	-5.100518000	-3.183963000	-0.770364000
H	-3.904652000	-4.157165000	2.657563000
H	-2.393941000	-4.219733000	1.751263000
H	-2.702783000	-2.867196000	2.843189000
N	5.048276000	-2.232477000	-0.371526000
N	6.055197000	0.453591000	0.510819000
C	4.868237000	-3.232291000	-1.412693000
H	4.303551000	-4.112392000	-1.067279000
H	4.358230000	-2.791684000	-2.271024000
H	5.854978000	-3.586485000	-1.737453000
C	5.809594000	-2.690441000	0.788504000
H	5.956155000	-1.871839000	1.492578000
H	5.274571000	-3.503264000	1.302872000
H	6.790402000	-3.066895000	0.474627000
C	7.158845000	0.004245000	-0.342243000
H	7.995190000	-0.339063000	0.275936000
H	6.834708000	-0.809232000	-0.987594000
H	7.507188000	0.836397000	-0.971236000
C	6.391475000	1.590811000	1.352163000
H	5.629236000	1.727241000	2.121264000
H	7.349167000	1.388303000	1.841999000
H	6.499417000	2.522135000	0.774858000
H	2.488585000	5.096864000	1.401172000
H	-0.187220000	5.208337000	1.155612000
N	-5.410672000	0.022032000	-0.523749000
O	-5.544378000	-0.961131000	-1.267699000
O	-6.342044000	0.664802000	-0.036353000
N	-4.355052000	2.921073000	-0.522446000
O	-5.226844000	2.805801000	-1.384478000
O	-4.071703000	3.986852000	0.050387000

E = –2146.4380 a. u.

2. IRC point, BÔB = 124.7°

Ground-state geometry, S_0:

F	-0.264076000	-0.017912000	-1.601162000
B	-1.273409000	1.069492000	0.856120000
B	0.407892000	-0.222825000	-0.379347000
O	-0.109728000	0.351416000	0.789889000
F	-1.567316000	1.499890000	2.164723000
C	-0.695306000	4.400597000	-0.815414000
C	-0.157675000	3.099354000	-0.438247000
C	1.211761000	2.817492000	-0.504536000
C	2.083981000	1.695809000	-0.396562000
C	3.481268000	1.821755000	-0.118219000
C	4.023075000	0.555494000	0.010639000
C	2.987690000	-0.404730000	-0.252949000
C	2.941330000	-1.846142000	-0.229214000
C	1.676789000	-2.430620000	0.020052000
C	1.320735000	-3.747352000	0.521619000
C	-0.026745000	-3.757451000	0.736866000
C	-0.557202000	-2.459421000	0.373149000
C	-1.935285000	-2.157922000	0.462846000
C	-2.788228000	-1.045764000	0.321289000
C	-4.241578000	-1.201514000	0.204760000
C	-4.808168000	0.068656000	0.137904000
C	-3.692142000	1.006863000	0.175490000
C	-3.631836000	2.388028000	-0.042244000
C	-2.389131000	3.025630000	-0.242747000
C	-2.058589000	4.352618000	-0.702731000
N	-1.233165000	2.328743000	-0.069864000
N	1.853405000	0.341595000	-0.479428000
N	0.505491000	-1.719456000	-0.071593000
N	-2.524874000	0.294812000	0.318311000
H	2.006717000	-4.547160000	0.746440000
H	-0.618419000	-4.567274000	1.142319000
H	1.781685000	3.719346000	-0.699449000
H	-2.518781000	-3.049534000	0.681984000
H	-4.537631000	2.966927000	-0.151199000
C	4.151407000	-2.773132000	-0.536260000
C	3.686624000	-3.857199000	-1.554210000

C	5.319462000	-2.045088000	-1.249777000
C	4.705229000	-3.452618000	0.740516000
H	2.884610000	-4.494109000	-1.185362000
H	3.339670000	-3.388458000	-2.481332000
H	4.536280000	-4.502943000	-1.798135000
H	5.997786000	-1.544238000	-0.564821000
H	5.924238000	-2.789859000	-1.774785000
H	4.966486000	-1.328006000	-1.997074000
H	5.562230000	-4.080133000	0.471935000
H	5.038090000	-2.706935000	1.463858000
H	3.963731000	-4.089650000	1.226609000
H	-2.770414000	5.131767000	-0.939095000
H	-0.092722000	5.227241000	-1.167977000
N	-4.863248000	-2.454570000	0.205416000
C	-6.148615000	-2.580400000	0.883698000
H	-6.284106000	-3.627092000	1.178380000
H	-6.150434000	-1.961218000	1.782227000
H	-7.006432000	-2.286838000	0.260072000
C	-4.728089000	-3.294177000	-0.985950000
H	-3.746050000	-3.149054000	-1.438636000
H	-4.831808000	-4.348456000	-0.706153000
H	-5.494588000	-3.068504000	-1.747197000
N	-6.160905000	0.359060000	-0.035237000
C	-6.721174000	1.580690000	0.524123000
H	-7.778430000	1.413277000	0.760675000
H	-6.200906000	1.842901000	1.447569000
H	-6.671076000	2.436076000	-0.170822000
C	-6.785630000	-0.050993000	-1.290696000
H	-6.352786000	-0.988271000	-1.638286000
H	-7.861140000	-0.197752000	-1.140335000
H	-6.644854000	0.706251000	-2.081077000
N	4.152532000	3.061868000	0.224575000
O	3.950985000	4.035976000	-0.506667000
O	4.857947000	3.064364000	1.229562000
N	5.352890000	0.333308000	0.538627000
O	5.445730000	-0.406305000	1.518558000
O	6.286383000	0.908579000	-0.015227000

$E = -2146.5128$ a. u.

Excited-state geometry, S_1:

F	-0.245213000	0.109574000	-1.201070000
B	-1.347460000	1.181993000	1.126989000
B	0.412201000	-0.127724000	0.039953000
O	-0.209818000	0.434185000	1.163659000
F	-1.829638000	1.604867000	2.373598000
C	-0.582758000	4.414871000	-0.647625000
C	-0.098766000	3.142379000	-0.133456000
C	1.276484000	2.869785000	-0.095436000
C	2.142485000	1.752685000	0.017278000
C	3.573063000	1.841908000	0.156538000
C	4.082405000	0.535242000	0.180046000
C	2.986413000	-0.372193000	-0.000391000
C	2.889131000	-1.801897000	-0.148641000
C	1.639212000	-2.376845000	0.172855000
C	1.269021000	-3.738572000	0.516651000
C	-0.063391000	-3.741687000	0.821629000
C	-0.573729000	-2.398064000	0.643969000
C	-1.938094000	-2.089407000	0.724970000
C	-2.794948000	-0.990108000	0.467863000
C	-4.219531000	-1.157687000	0.183318000
C	-4.773878000	0.135510000	-0.060057000
C	-3.695212000	1.080603000	0.130570000
C	-3.589683000	2.455877000	-0.148828000
C	-2.344604000	3.085774000	-0.191032000
C	-1.950805000	4.374137000	-0.694699000
N	-1.214492000	2.397963000	0.175230000
N	1.853814000	0.409863000	-0.036522000
N	0.498623000	-1.625887000	0.278255000
N	-2.579229000	0.357317000	0.476020000
H	1.940471000	-4.578934000	0.578821000
H	-0.656537000	-4.587124000	1.144424000
H	1.858858000	3.767113000	-0.268044000
H	-2.504923000	-2.982810000	0.957695000
H	-4.457759000	3.028763000	-0.437384000
C	3.989122000	-2.711923000	-0.753812000
C	3.322764000	-3.616186000	-1.831712000
C	5.079314000	-1.932078000	-1.528160000
C	4.675355000	-3.589564000	0.317994000
H	2.546478000	-4.271241000	-1.439596000
H	2.874790000	-3.002286000	-2.620496000
H	4.092290000	-4.246635000	-2.288130000
H	5.875821000	-1.562053000	-0.889975000
H	5.550199000	-2.615630000	-2.240541000
H	4.661477000	-1.100456000	-2.102881000
H	5.446240000	-4.206025000	-0.157907000
H	5.150709000	-2.964380000	1.074745000
H	3.971188000	-4.260240000	0.816210000
H	-2.622620000	5.132573000	-1.073221000
H	0.066602000	5.211098000	-0.985153000
N	-4.930230000	-2.332633000	0.155170000
C	-6.202354000	-2.457450000	0.857114000
H	-6.093136000	-3.182456000	1.674519000
H	-6.495412000	-1.497550000	1.279218000
H	-6.989815000	-2.818786000	0.185453000
C	-4.421434000	-3.571239000	-0.422343000
H	-3.535700000	-3.373213000	-1.022439000
H	-4.185307000	-4.316685000	0.347263000
H	-5.201616000	-3.994948000	-1.068168000
N	-6.043596000	0.403659000	-0.482723000
C	-6.645399000	1.719350000	-0.305804000
H	-7.722103000	1.587337000	-0.159108000
H	-6.233315000	2.205003000	0.577715000
H	-6.497573000	2.359849000	-1.185477000
C	-6.761358000	-0.461709000	-1.414812000
H	-6.140827000	-1.307688000	-1.702733000
H	-7.697748000	-0.829051000	-0.979175000
H	-7.006887000	0.115846000	-2.314457000
N	4.296718000	3.029758000	0.464735000
O	3.890048000	4.109148000	-0.020313000
O	5.279482000	2.934510000	1.211920000
N	5.434445000	0.189386000	0.477323000
O	5.616003000	-0.726031000	1.301512000
O	6.337108000	0.803954000	-0.098119000

$E = -2146.4229$ a. u.

Excited-state geometry, T_1:

F	-0.272839000	0.026573000	-1.255632000
B	-1.318813000	1.222933000	1.083297000
B	0.390562000	-0.148563000	-0.018050000
O	-0.170913000	0.465699000	1.100042000
F	-1.681317000	1.752184000	2.330692000
C	-0.573981000	4.374733000	-0.912074000
C	-0.074924000	3.134428000	-0.321757000

C	1.286369000	2.846287000	-0.328062000
C	2.160004000	1.707464000	-0.141625000
C	3.569078000	1.781292000	0.007416000
C	4.064005000	0.462194000	0.101182000
C	2.969551000	-0.419601000	-0.052191000
C	2.841899000	-1.865649000	-0.163339000
C	1.575297000	-2.415724000	0.256333000
C	1.218057000	-3.745029000	0.713619000
C	-0.113680000	-3.723873000	1.021565000
C	-0.624028000	-2.398606000	0.750428000
C	-1.965667000	-2.057721000	0.847086000
C	-2.807438000	-0.929892000	0.555212000
C	-4.185057000	-1.135983000	0.231246000
C	-4.753644000	0.141122000	-0.023786000
C	-3.675285000	1.093092000	0.142319000
C	-3.575267000	2.453312000	-0.213017000
C	-2.315881000	3.068674000	-0.327203000
C	-1.937441000	4.326680000	-0.929309000
N	-1.189066000	2.412493000	0.064626000
N	1.846879000	0.371903000	-0.146585000
N	0.462902000	-1.653918000	0.297847000
N	-2.544066000	0.412752000	0.517737000
H	1.894074000	-4.576881000	0.825144000
H	-0.709495000	-4.541635000	1.404781000
H	1.873497000	3.711644000	-0.613478000
H	-2.582364000	-2.909098000	1.132353000
H	-4.448672000	3.013156000	-0.514800000
C	3.863863000	-2.793108000	-0.856422000
C	3.089232000	-3.691836000	-1.867303000
C	4.902995000	-2.023363000	-1.707331000
C	4.617954000	-3.686868000	0.160977000
H	2.355335000	-4.345500000	-1.395380000
H	2.567520000	-3.077944000	-2.609293000
H	3.805953000	-4.327950000	-2.396307000
H	5.717956000	-1.610335000	-1.118908000
H	5.359883000	-2.723561000	-2.412780000
H	4.440808000	-1.222233000	-2.291577000
H	5.321014000	-4.328866000	-0.381552000
H	5.179623000	-3.076198000	0.868579000
H	3.942951000	-4.334780000	0.724353000
H	-2.622706000	5.054447000	-1.342164000
H	0.064688000	5.148274000	-1.317404000
N	-4.791660000	-2.402154000	0.238180000
C	-6.105444000	-2.539681000	0.855390000
H	-6.200603000	-3.552377000	1.264783000
H	-6.201794000	-1.825680000	1.675374000
H	-6.940941000	-2.382727000	0.155539000
C	-4.534597000	-3.326292000	-0.864360000
H	-3.529661000	-3.166814000	-1.258517000
H	-4.608887000	-4.359283000	-0.504972000
H	-5.253526000	-3.208761000	-1.694357000
N	-6.055023000	0.399417000	-0.400067000
C	-6.681670000	1.677670000	-0.104975000
H	-7.756553000	1.521129000	0.040200000
H	-6.265056000	2.096492000	0.812599000
H	-6.559647000	2.404188000	-0.924250000
C	-6.636376000	-0.300477000	-1.541806000
H	-6.108138000	-1.232680000	-1.726602000
H	-7.693838000	-0.518373000	-1.356282000
H	-6.562795000	0.318813000	-2.450069000
N	4.320980000	2.979239000	0.251898000
O	3.983857000	4.011437000	-0.352488000
O	5.246213000	2.919348000	1.062641000
N	5.421665000	0.099490000	0.376552000
O	5.606092000	-0.823113000	1.184091000
O	6.316529000	0.708985000	-0.210208000

$E = -2146.4403$ a. u.

3. IRC point, BÔB = 143.9°

Ground-state geometry, S_0:

F	-0.001141000	-0.280720000	1.907885000
B	1.287195000	1.056474000	-0.784941000
B	-0.459307000	-0.292815000	0.573844000
O	0.181097000	0.353133000	-0.457351000
F	1.342875000	1.333746000	-2.168833000
C	0.752967000	4.417641000	0.793653000
C	0.218434000	3.113302000	0.412576000
C	-1.152764000	2.805389000	0.495283000
C	-2.056007000	1.693386000	0.389543000
C	-3.449695000	1.845782000	0.089491000
C	-4.020974000	0.589303000	-0.037697000
C	-3.011716000	-0.393483000	0.250017000
C	-2.969869000	-1.841028000	0.202929000
C	-1.708307000	-2.436677000	-0.061191000
C	-1.340119000	-3.731161000	-0.615979000
C	0.010561000	-3.719597000	-0.826187000
C	0.534529000	-2.431191000	-0.407807000
C	1.920944000	-2.115563000	-0.483324000
C	2.805241000	-1.019163000	-0.344733000
C	4.257922000	-1.194753000	-0.230515000
C	4.849624000	0.068026000	-0.174316000
C	3.752530000	1.031422000	-0.233654000
C	3.700460000	2.421297000	-0.036363000
C	2.460112000	3.072147000	0.169817000
C	2.116608000	4.389950000	0.654883000
N	1.306490000	2.374897000	0.013747000
N	-1.878774000	0.332080000	0.507265000
N	-0.542303000	-1.734714000	0.069943000
N	2.580535000	0.329663000	-0.355249000
H	-2.018709000	-4.525623000	-0.881411000
H	0.606346000	-4.506251000	-1.269774000
H	-1.719717000	3.707228000	0.702613000
H	2.497778000	-3.011479000	-0.706743000
H	4.611153000	2.994660000	0.061128000
C	-4.180140000	-2.759369000	0.515488000
C	-3.701492000	-3.844704000	1.525411000
C	-5.335215000	-2.022292000	1.238317000
C	-4.747582000	-3.437780000	-0.755080000
H	-2.912834000	-4.485313000	1.132610000
H	-3.327066000	-3.377927000	2.442669000
H	-4.549150000	-4.484818000	1.790690000
H	-6.004044000	-1.500846000	0.558925000
H	-5.952158000	-2.763400000	1.754550000
H	-4.969003000	-1.320273000	1.993512000
H	-5.601360000	-4.065756000	-0.477529000
H	-5.088103000	-2.691092000	-1.474237000
H	-4.010976000	-4.074979000	-1.248489000
H	2.818770000	5.177519000	0.892476000
H	0.150505000	5.235064000	1.167595000
N	4.860387000	-2.455938000	-0.212313000
C	6.146579000	-2.615354000	-0.881075000
H	6.260546000	-3.666888000	-1.167525000
H	6.168221000	-2.002567000	-1.783704000
H	7.006790000	-2.336825000	-0.253817000

C	4.693172000	-3.289790000	0.979203000
H	3.714101000	-3.113880000	1.427052000
H	4.765728000	-4.347319000	0.701720000
H	5.462909000	-3.086729000	1.743427000
N	6.206229000	0.328410000	0.021596000
C	6.797051000	1.547081000	-0.511941000
H	7.858408000	1.368772000	-0.719614000
H	6.306838000	1.822873000	-1.447891000
H	6.736796000	2.397093000	0.188913000
C	6.797412000	-0.096767000	1.289606000
H	6.341892000	-1.027448000	1.624860000
H	7.873122000	-0.260689000	1.160973000
H	6.652123000	0.660686000	2.078862000
N	-4.105934000	3.097475000	-0.241810000
O	-3.878490000	4.065098000	0.490945000
O	-4.832939000	3.115117000	-1.231203000
N	-5.356907000	0.384195000	-0.555949000
O	-5.468689000	-0.362010000	-1.529271000
O	-6.278931000	0.968896000	0.007310000

E = –2146.4966 a. u.

Excited-state geometry, S_1:

F	0.109578000	-0.038056000	1.457429000
B	1.358653000	1.175049000	-1.100560000
B	-0.446355000	-0.182240000	0.147234000
O	0.272973000	0.428193000	-0.849617000
F	1.647810000	1.492022000	-2.429464000
C	0.664014000	4.417974000	0.672942000
C	0.173055000	3.140604000	0.169500000
C	-1.204085000	2.847245000	0.186536000
C	-2.103492000	1.744464000	0.071663000
C	-3.529837000	1.865931000	-0.090100000
C	-4.071578000	0.570181000	-0.140695000
C	-3.003566000	-0.367472000	0.048286000
C	-2.921889000	-1.808986000	0.150060000
C	-1.669961000	-2.392355000	-0.161572000
C	-1.287905000	-3.740487000	-0.553732000
C	0.052178000	-3.728799000	-0.829270000
C	0.559925000	-2.388769000	-0.596642000
C	1.925026000	-2.060748000	-0.681627000
C	2.806900000	-0.962536000	-0.457887000
C	4.233187000	-1.146502000	-0.183239000
C	4.823105000	0.144697000	-0.007321000
C	3.765911000	1.107725000	-0.228231000
C	3.673124000	2.494347000	0.024568000
C	2.429631000	3.131244000	0.104044000
C	2.034025000	4.407614000	0.643501000
N	1.293640000	2.436183000	-0.213825000
N	-1.862991000	0.393069000	0.129888000
N	-0.525762000	-1.647249000	-0.209466000
N	2.635773000	0.389888000	-0.528457000
H	-1.954900000	-4.579031000	-0.670217000
H	0.649306000	-4.560077000	-1.180672000
H	-1.776135000	3.743741000	0.396609000
H	2.492103000	-2.956227000	-0.911262000
H	4.553443000	3.065735000	0.278177000
C	-4.051954000	-2.725622000	0.681201000
C	-3.434128000	-3.690490000	1.734581000
C	-5.152647000	-1.961065000	1.455427000
C	-4.714928000	-3.541474000	-0.452486000
H	-2.658022000	-4.340562000	1.333622000
H	-2.998705000	-3.122391000	2.563580000
H	-4.227308000	-4.327461000	2.138589000
H	-5.921425000	-1.548145000	0.809346000
H	-5.658382000	-2.666001000	2.121655000
H	-4.738350000	-1.163664000	2.079160000
H	-5.509453000	-4.168395000	-0.032338000
H	-5.156194000	-2.874654000	-1.194363000
H	-4.002039000	-4.197693000	-0.957825000
H	2.706336000	5.177010000	0.998417000
H	0.020919000	5.198491000	1.056373000
N	4.914217000	-2.336122000	-0.105386000
C	6.178720000	-2.527749000	-0.805324000
H	6.046823000	-3.289444000	-1.585162000
H	6.493590000	-1.597432000	-1.275324000
H	6.960633000	-2.875372000	-0.120057000
C	4.377817000	-3.529564000	0.538898000
H	3.492828000	-3.280321000	1.120584000
H	4.130594000	-4.311711000	-0.189572000
H	5.145873000	-3.931613000	1.212736000
N	6.104076000	0.401107000	0.388320000
C	6.724740000	1.698928000	0.154752000
H	7.797145000	1.544453000	-0.001397000
H	6.307761000	2.158418000	-0.740524000
H	6.599071000	2.374122000	1.011764000
C	6.811023000	-0.434567000	1.355173000
H	6.176668000	-1.255053000	1.683894000
H	7.738033000	-0.838300000	0.931865000
H	7.071967000	0.177475000	2.227374000
N	-4.223069000	3.072104000	-0.400894000
O	-3.798302000	4.137812000	0.095973000
O	-5.198097000	3.004159000	-1.160514000
N	-5.428338000	0.265192000	-0.467858000
O	-5.617609000	-0.614448000	-1.326681000
O	-6.324222000	0.876507000	0.119655000

E = –2146.4513 a. u.

Excited-state geometry, T_1:

F	0.135685000	-0.143813000	1.501022000
B	1.325872000	1.199099000	-1.064383000
B	-0.432795000	-0.213872000	0.200253000
O	0.235607000	0.441215000	-0.793745000
F	1.495064000	1.607911000	-2.389957000
C	0.679941000	4.357824000	0.946075000
C	0.161414000	3.124909000	0.351002000
C	-1.199226000	2.821427000	0.420051000
C	-2.116402000	1.706435000	0.213804000
C	-3.517658000	1.826043000	0.023613000
C	-4.055879000	0.523381000	-0.094494000
C	-2.998141000	-0.398266000	0.083890000
C	-2.900858000	-1.854393000	0.151680000
C	-1.631957000	-2.422853000	-0.249097000
C	-1.270577000	-3.739605000	-0.745364000
C	0.070894000	-3.719108000	-1.013045000
C	0.587600000	-2.404010000	-0.692430000
C	1.931904000	-2.055677000	-0.792170000
C	2.806703000	-0.932150000	-0.543025000
C	4.184994000	-1.157356000	-0.228985000
C	4.793487000	0.113808000	-0.042912000
C	3.740244000	1.090175000	-0.241276000
C	3.662421000	2.462640000	0.086411000
C	2.409919000	3.092590000	0.236714000
C	2.043424000	4.332793000	0.887195000
N	1.271755000	2.442614000	-0.120332000

N	-1.861641000	0.359951000	0.231404000
N	-0.510773000	-1.679109000	-0.232257000
N	2.592755000	0.421028000	-0.574656000
H	-1.948821000	-4.560960000	-0.910922000
H	0.666658000	-4.527891000	-1.415172000
H	-1.765401000	3.679168000	0.765884000
H	2.540807000	-2.916760000	-1.068303000
H	4.549803000	3.015301000	0.360276000
C	-3.967579000	-2.779726000	0.771730000
C	-3.252088000	-3.736235000	1.772494000
C	-5.017707000	-2.014479000	1.612035000
C	-4.702520000	-3.615407000	-0.307690000
H	-2.517193000	-4.388240000	1.299437000
H	-2.742927000	-3.165841000	2.556634000
H	-4.002081000	-4.375494000	2.249194000
H	-5.794224000	-1.552221000	1.008417000
H	-5.523919000	-2.728287000	2.268558000
H	-4.555731000	-1.252764000	2.246922000
H	-5.443905000	-4.256862000	0.181516000
H	-5.218650000	-2.964988000	-1.014814000
H	-4.020522000	-4.260943000	-0.865860000
H	2.736865000	5.061208000	1.285042000
H	0.055815000	5.113117000	1.405066000
N	4.758136000	-2.438204000	-0.184334000
C	6.064378000	-2.637737000	-0.800251000
H	6.129950000	-3.669154000	-1.166407000
H	6.176278000	-1.961591000	-1.649782000
H	6.907311000	-2.474115000	-0.110828000
C	4.481608000	-3.307321000	0.957741000
H	3.484596000	-3.101414000	1.350317000
H	4.522726000	-4.356288000	0.641933000
H	5.209569000	-3.176167000	1.777721000
N	6.105606000	0.351084000	0.311751000
C	6.758170000	1.601822000	-0.040004000
H	7.828387000	1.416501000	-0.185735000
H	6.343729000	1.992404000	-0.971015000
H	6.658032000	2.364077000	0.749419000
C	6.669172000	-0.302958000	1.489365000
H	6.124851000	-1.216820000	1.714892000
H	7.723484000	-0.547015000	1.319550000
H	6.601875000	0.359551000	2.367278000
N	-4.221398000	3.049446000	-0.239709000
O	-3.855893000	4.070876000	0.365699000
O	-5.134449000	3.020370000	-1.065723000
N	-5.418019000	0.211848000	-0.413564000
O	-5.610370000	-0.682224000	-1.250071000
O	-6.307212000	0.832197000	0.169513000

E = –2146.4297 a. u.

4. IRC point, BÔB = 170.2°

Ground-state geometry, S_0

F	-0.165842000	-0.536792000	2.067265000
B	1.353113000	0.988703000	-0.728791000
B	-0.435408000	-0.401829000	0.688623000
O	0.376830000	0.324194000	-0.094338000
F	1.196910000	1.131872000	-2.125932000
C	0.843417000	4.362981000	0.813210000
C	0.309060000	3.071155000	0.388109000
C	-1.061283000	2.744137000	0.485992000
C	-1.975865000	1.633379000	0.370952000
C	-3.374769000	1.795010000	0.088640000
C	-3.958560000	0.542379000	-0.036105000
C	-2.955478000	-0.447997000	0.238218000
C	-2.913447000	-1.898596000	0.194546000
C	-1.654553000	-2.494339000	-0.086828000
C	-1.273229000	-3.791745000	-0.623438000
C	0.078700000	-3.768498000	-0.832968000
C	0.592725000	-2.468154000	-0.432057000
C	1.984130000	-2.150247000	-0.491589000
C	2.883092000	-1.060254000	-0.367548000
C	4.331501000	-1.249230000	-0.226568000
C	4.934002000	0.009338000	-0.170271000
C	3.848751000	0.987665000	-0.264265000
C	3.796665000	2.378124000	-0.049862000
C	2.556332000	3.041543000	0.140285000
C	2.208058000	4.341334000	0.676971000
N	1.403312000	2.364943000	-0.056383000
N	-1.817533000	0.268606000	0.480443000
N	-0.495885000	-1.777573000	0.024173000
N	2.683938000	0.293104000	-0.417500000
H	-1.942412000	-4.597239000	-0.880677000
H	0.680488000	-4.555277000	-1.268409000
H	-1.629157000	3.639316000	0.721388000
H	2.558395000	-3.054265000	-0.688869000
H	4.708890000	2.941423000	0.084909000
C	-4.119541000	-2.813569000	0.523229000
C	-3.633891000	-3.894952000	1.533855000
C	-5.269887000	-2.073126000	1.249421000
C	-4.692308000	-3.495800000	-0.742831000
H	-2.846963000	-4.535701000	1.137591000
H	-3.253889000	-3.424737000	2.446940000
H	-4.479452000	-4.534975000	1.805995000
H	-5.940412000	-1.551814000	0.571439000
H	-5.885826000	-2.812031000	1.770032000
H	-4.898618000	-1.370323000	2.001406000
H	-5.543167000	-4.125429000	-0.460086000
H	-5.038123000	-2.750797000	-1.461470000
H	-3.955875000	-4.131832000	-1.238427000
H	2.908065000	5.118970000	0.950615000
H	0.241227000	5.166776000	1.216038000
N	4.923725000	-2.515863000	-0.198039000
C	6.209085000	-2.688422000	-0.864615000
H	6.316225000	-3.742196000	-1.145513000
H	6.235710000	-2.080747000	-1.770669000
H	7.071129000	-2.412618000	-0.238408000
C	4.745351000	-3.347172000	0.993558000
H	3.767399000	-3.160270000	1.439499000
H	4.806698000	-4.405595000	0.716514000
H	5.515805000	-3.152949000	1.759419000
N	6.287142000	0.262529000	0.041915000
C	6.886866000	1.478761000	-0.486107000
H	7.948153000	1.295777000	-0.690003000
H	6.401807000	1.759462000	-1.423200000
H	6.827772000	2.327100000	0.216647000
C	6.867976000	-0.172791000	1.310699000
H	6.402513000	-1.100684000	1.639570000
H	7.942929000	-0.345176000	1.187469000
H	6.724439000	0.583006000	2.101776000
N	-4.033245000	3.049033000	-0.227862000
O	-3.800258000	4.010986000	0.510485000
O	-4.770935000	3.072821000	-1.209363000
N	-5.298110000	0.339923000	-0.543315000
O	-5.416563000	-0.408868000	-1.514285000
O	-6.216380000	0.922559000	0.027792000

E = –2146.4835 a. u.

Excited-state geometry, S_1:

F	-0.115780000	-0.361037000	1.915064000
B	1.383802000	1.042310000	-0.868332000
B	-0.433062000	-0.333319000	0.529806000
O	0.422696000	0.337036000	-0.270174000
F	1.377153000	1.158347000	-2.267757000
C	0.782007000	4.390790000	0.686901000
C	0.279923000	3.090442000	0.250101000
C	-1.089430000	2.762914000	0.337524000
C	-2.006908000	1.659329000	0.214700000
C	-3.417899000	1.807353000	-0.032574000
C	-3.986537000	0.522049000	-0.109873000
C	-2.956089000	-0.437064000	0.148292000
C	-2.891743000	-1.888634000	0.204722000
C	-1.633172000	-2.477826000	-0.076346000
C	-1.239456000	-3.798963000	-0.546070000
C	0.108886000	-3.769245000	-0.780508000
C	0.613695000	-2.444197000	-0.452389000
C	1.983855000	-2.108659000	-0.542388000
C	2.892702000	-1.015948000	-0.360250000
C	4.331092000	-1.193858000	-0.147861000
C	4.932184000	0.103843000	-0.040255000
C	3.871853000	1.066735000	-0.250783000
C	3.780883000	2.467964000	-0.053088000
C	2.538466000	3.110101000	0.054376000
C	2.148733000	4.399696000	0.577913000
N	1.394727000	2.404895000	-0.181730000
N	-1.813862000	0.301498000	0.318951000
N	-0.485423000	-1.742048000	-0.026133000
N	2.734675000	0.334645000	-0.465552000
H	-1.902305000	-4.625226000	-0.747063000
H	0.712094000	-4.573070000	-1.182231000
H	-1.662805000	3.655395000	0.562337000
H	2.541696000	-3.006114000	-0.791131000
H	4.670015000	3.050252000	0.137742000
C	-4.060885000	-2.807001000	0.629968000
C	-3.511909000	-3.834274000	1.660992000
C	-5.183533000	-2.053232000	1.381177000
C	-4.677105000	-3.557260000	-0.573635000
H	-2.733358000	-4.480906000	1.257633000
H	-3.098959000	-3.317927000	2.533970000
H	-4.334648000	-4.472294000	1.999212000
H	-5.897156000	-1.574104000	0.717431000
H	-5.752721000	-2.778589000	1.970042000
H	-4.782110000	-1.309827000	2.076159000
H	-5.505088000	-4.184866000	-0.225286000
H	-5.064070000	-2.850728000	-1.308767000
H	-3.949477000	-4.206414000	-1.067020000
H	2.825336000	5.190986000	0.871121000
H	0.151657000	5.177881000	1.078182000
N	5.009398000	-2.382884000	-0.062849000
C	6.256365000	-2.597176000	-0.786911000
H	6.103459000	-3.378365000	-1.543198000
H	6.562234000	-1.680607000	-1.288678000
H	7.053423000	-2.929520000	-0.111653000
C	4.483420000	-3.556123000	0.626470000
H	3.609586000	-3.288331000	1.216579000
H	4.220455000	-4.357807000	-0.074534000
H	5.263836000	-3.939895000	1.296732000
N	6.228959000	0.366632000	0.298019000
C	6.842364000	1.654287000	-0.001291000
H	7.909310000	1.493181000	-0.185502000
H	6.397174000	2.081940000	-0.898836000
H	6.743534000	2.360240000	0.834310000
C	6.962689000	-0.433978000	1.274796000
H	6.336268000	-1.238883000	1.653875000
H	7.875491000	-0.857577000	0.840277000
H	7.250789000	0.209614000	2.115292000
N	-4.069037000	3.022952000	-0.399846000
O	-3.658052000	4.085562000	0.111063000
O	-4.995965000	2.963466000	-1.217470000
N	-5.331333000	0.252029000	-0.517388000
O	-5.491818000	-0.601238000	-1.405573000
O	-6.243730000	0.867089000	0.038234000

E = –2146.4395 a. u.

Excited-state geometry, T_1:

F	-0.162607000	-0.408492000	1.939392000
B	1.283486000	1.111122000	-0.851349000
B	-0.493968000	-0.307353000	0.569752000
O	0.328331000	0.388425000	-0.234009000
F	1.146384000	1.319669000	-2.233760000
C	0.773217000	4.379548000	0.963865000
C	0.225622000	3.132375000	0.424628000
C	-1.128395000	2.809628000	0.553301000
C	-2.073389000	1.705666000	0.319985000
C	-3.448306000	1.870973000	0.013040000
C	-4.026837000	0.587060000	-0.130598000
C	-3.024352000	-0.370451000	0.145445000
C	-2.964764000	-1.834250000	0.174417000
C	-1.687373000	-2.421804000	-0.183080000
C	-1.327457000	-3.707135000	-0.759699000
C	0.023976000	-3.690788000	-0.972716000
C	0.552280000	-2.404981000	-0.552945000
C	1.902036000	-2.063142000	-0.670834000
C	2.816187000	-0.952623000	-0.479271000
C	4.215520000	-1.174836000	-0.257942000
C	4.840053000	0.095520000	-0.147048000
C	3.781985000	1.077438000	-0.301641000
C	3.717214000	2.463255000	-0.015308000
C	2.473537000	3.108275000	0.178397000
C	2.131758000	4.362872000	0.821922000
N	1.318707000	2.456562000	-0.098864000
N	-1.883323000	0.348872000	0.397675000
N	-0.556321000	-1.709066000	-0.056397000
N	2.618196000	0.400173000	-0.536483000
H	-2.011684000	-4.499896000	-1.016595000
H	0.618624000	-4.475568000	-1.421589000
H	-1.683833000	3.663147000	0.927125000
H	2.488147000	-2.932129000	-0.973143000
H	4.619415000	3.021350000	0.191607000
C	-4.094455000	-2.748928000	0.680109000
C	-3.471202000	-3.770977000	1.676897000
C	-5.168968000	-1.979132000	1.482622000
C	-4.782444000	-3.516450000	-0.478206000
H	-2.726647000	-4.420870000	1.214727000
H	-2.994924000	-3.254554000	2.516951000
H	-4.266291000	-4.409417000	2.075100000
H	-5.865248000	-1.432165000	0.851860000
H	-5.766512000	-2.701889000	2.046364000
H	-4.718820000	-1.288166000	2.201739000
H	-5.582408000	-4.141390000	-0.065882000
H	-5.219494000	-2.823879000	-1.198049000

H	-4.087599000	-4.173344000	-1.006705000
H	2.839646000	5.107100000	1.160575000
H	0.173542000	5.146205000	1.436365000
N	4.785804000	-2.457195000	-0.226457000
C	6.061540000	-2.665437000	-0.900838000
H	6.118435000	-3.707974000	-1.235049000
H	6.123112000	-2.016925000	-1.776620000
H	6.936652000	-2.470230000	-0.261606000
C	4.572467000	-3.301105000	0.948177000
H	3.591668000	-3.097619000	1.381083000
H	4.611465000	-4.356516000	0.654893000
H	5.334774000	-3.142523000	1.731249000
N	6.177870000	0.328949000	0.116812000
C	6.807454000	1.567731000	-0.310901000
H	7.870533000	1.381348000	-0.500657000
H	6.347605000	1.921929000	-1.235416000
H	6.741475000	2.360659000	0.452271000
C	6.781492000	-0.261212000	1.308810000
H	6.263375000	-1.177611000	1.582270000
H	7.836223000	-0.490747000	1.122675000
H	6.720673000	0.433983000	2.162214000
N	-4.080394000	3.113965000	-0.333210000
O	-3.716469000	4.136299000	0.270363000
O	-4.932589000	3.097840000	-1.221963000
N	-5.371503000	0.325519000	-0.557240000
O	-5.530221000	-0.552932000	-1.415733000
O	-6.279942000	0.970525000	-0.034263000

E = –2146.4145 a. u.

5. Transition state, BÔB = 174.9°

Ground-state geometry, S_0:

F	-0.269772000	-0.507447000	2.067896000
B	1.288027000	1.046934000	-0.735964000
B	-0.502094000	-0.346198000	0.684945000
O	0.352586000	0.393799000	-0.029347000
F	1.091345000	1.164495000	-2.130016000
C	0.785725000	4.426778000	0.795664000
C	0.250527000	3.138124000	0.362262000
C	-1.119117000	2.808584000	0.463502000
C	-2.033628000	1.697280000	0.345772000
C	-3.434006000	1.859090000	0.069112000
C	-4.018769000	0.606639000	-0.054823000
C	-3.015303000	-0.383783000	0.214375000
C	-2.973554000	-1.834559000	0.173632000
C	-1.715082000	-2.429008000	-0.111444000
C	-1.331916000	-3.728426000	-0.640298000
C	0.020085000	-3.704297000	-0.850124000
C	0.532736000	-2.401038000	-0.456380000
C	1.924691000	-2.084525000	-0.512598000
C	2.824936000	-0.995021000	-0.392528000
C	4.272200000	-1.185432000	-0.244703000
C	4.875322000	0.072719000	-0.188567000
C	3.791092000	1.052486000	-0.290369000
C	3.738280000	2.442455000	-0.071568000
C	2.497801000	3.107578000	0.114310000
C	2.150317000	4.404435000	0.660154000
N	1.344173000	2.436313000	-0.091721000
N	-1.875083000	0.332198000	0.449492000
N	-0.557508000	-1.707509000	-0.008551000
N	2.628545000	0.358437000	-0.452826000
H	-1.999725000	-4.536687000	-0.892567000
H	0.622763000	-4.492748000	-1.281317000
H	-1.687521000	3.702002000	0.704510000
H	2.498409000	-2.990338000	-0.703058000
H	4.650287000	3.003957000	0.072050000
C	-4.178922000	-2.749406000	0.506298000
C	-3.692309000	-3.829693000	1.517593000
C	-5.328842000	-2.008574000	1.232890000
C	-4.752910000	-3.433061000	-0.758526000
H	-2.905390000	-4.470604000	1.121610000
H	-3.311987000	-3.358514000	2.430021000
H	-4.537604000	-4.469695000	1.790596000
H	-6.000494000	-1.488578000	0.555001000
H	-5.943709000	-2.747145000	1.755229000
H	-4.957043000	-1.304606000	1.983524000
H	-5.603302000	-4.062637000	-0.474239000
H	-5.099738000	-2.688835000	-1.477517000
H	-4.016705000	-4.069345000	-1.254232000
H	2.851196000	5.179055000	0.939979000
H	0.184112000	5.228615000	1.203200000
N	4.863236000	-2.452729000	-0.213922000
C	6.148353000	-2.627230000	-0.880311000
H	6.254709000	-3.681435000	-1.159940000
H	6.175381000	-2.020757000	-1.787181000
H	7.010754000	-2.351377000	-0.254554000
C	4.683342000	-3.283293000	0.977918000
H	3.705666000	-3.094499000	1.423698000
H	4.742845000	-4.341941000	0.701234000
H	5.454009000	-3.090311000	1.743895000
N	6.227499000	0.325981000	0.026388000
C	6.828608000	1.541223000	-0.502042000
H	7.889704000	1.357034000	-0.705921000
H	6.343830000	1.822117000	-1.439207000
H	6.770403000	2.389859000	0.200374000
C	6.807698000	-0.111047000	1.294715000
H	6.340522000	-1.038218000	1.623129000
H	7.882336000	-0.285307000	1.171380000
H	6.665531000	0.644646000	2.086105000
N	-4.093817000	3.112823000	-0.245118000
O	-3.860580000	4.074238000	0.493794000
O	-4.833023000	3.136776000	-1.225528000
N	-5.359196000	0.403702000	-0.559799000
O	-5.478435000	-0.345495000	-1.530414000
O	-6.277062000	0.985765000	0.012403000

E = –2146.4829 a. u.

Excited-state geometry, S_1:

F	-0.233868000	-0.397835000	2.000796000
B	1.309513000	1.082046000	-0.782940000
B	-0.506575000	-0.308077000	0.612546000
O	0.375657000	0.379659000	-0.138632000
F	1.235322000	1.189606000	-2.184583000
C	0.758312000	4.430322000	0.778912000
C	0.241187000	3.135360000	0.341383000
C	-1.127085000	2.811597000	0.441474000
C	-2.053288000	1.715237000	0.295804000
C	-3.459514000	1.881796000	0.037381000
C	-4.040808000	0.604473000	-0.069659000
C	-3.023231000	-0.369610000	0.182179000
C	-2.973217000	-1.822711000	0.204019000
C	-1.718375000	-2.416450000	-0.087266000
C	-1.336755000	-3.723653000	-0.601980000

C	0.011517000	-3.695819000	-0.838426000
C	0.527692000	-2.386744000	-0.467160000
C	1.900596000	-2.059831000	-0.566637000
C	2.824158000	-0.981016000	-0.379066000
C	4.267478000	-1.167721000	-0.211457000
C	4.875593000	0.126310000	-0.091060000
C	3.814620000	1.097546000	-0.251654000
C	3.734203000	2.497697000	-0.034275000
C	2.497378000	3.143997000	0.106522000
C	2.122628000	4.432876000	0.645386000
N	1.345542000	2.447766000	-0.112451000
N	-1.875463000	0.353632000	0.380776000
N	-0.565142000	-1.692337000	-0.009533000
N	2.670469000	0.372892000	-0.446910000
H	-2.006899000	-4.536640000	-0.831119000
H	0.607196000	-4.488892000	-1.271583000
H	-1.694422000	3.702425000	0.687180000
H	2.444356000	-2.956213000	-0.847509000
H	4.630622000	3.073067000	0.142874000
C	-4.153179000	-2.739308000	0.599773000
C	-3.618399000	-3.797865000	1.606267000
C	-5.270741000	-1.992018000	1.364640000
C	-4.772060000	-3.454463000	-0.623755000
H	-2.844845000	-4.441150000	1.187863000
H	-3.203048000	-3.308457000	2.493467000
H	-4.448614000	-4.436165000	1.925205000
H	-5.972846000	-1.484015000	0.710067000
H	-5.853818000	-2.725924000	1.928864000
H	-4.863705000	-1.274349000	2.083048000
H	-5.608153000	-4.080824000	-0.293020000
H	-5.147957000	-2.727465000	-1.344368000
H	-4.049373000	-4.100413000	-1.128432000
H	2.808219000	5.219720000	0.929495000
H	0.139119000	5.219168000	1.184246000
N	4.945866000	-2.358427000	-0.170443000
C	6.178275000	-2.556230000	-0.924356000
H	6.009985000	-3.322385000	-1.692495000
H	6.474327000	-1.629238000	-1.412455000
H	6.988917000	-2.901628000	-0.272203000
C	4.427865000	-3.552822000	0.487754000
H	3.574477000	-3.300784000	1.113711000
H	4.137402000	-4.324411000	-0.235924000
H	5.222855000	-3.969255000	1.119980000
N	6.183674000	0.374778000	0.215835000
C	6.793865000	1.666175000	-0.072848000
H	7.855719000	1.506224000	-0.285563000
H	6.328916000	2.112488000	-0.951069000
H	6.717238000	2.356197000	0.778367000
C	6.930130000	-0.438865000	1.172394000
H	6.312650000	-1.255852000	1.539959000
H	7.842270000	-0.846582000	0.721872000
H	7.220516000	0.189808000	2.023609000
N	-4.094181000	3.110860000	-0.317618000
O	-3.683117000	4.158973000	0.221195000
O	-5.006988000	3.075312000	-1.151767000
N	-5.384814000	0.357895000	-0.494517000
O	-5.547837000	-0.476872000	-1.399375000
O	-6.294601000	0.974214000	0.064214000

E = –2146.4388 a. u.

Excited-state geometry, T_1:

F	-0.230409000	-0.456450000	2.017729000
B	1.272781000	1.091827000	-0.789272000
B	-0.507572000	-0.323681000	0.641566000
O	0.345047000	0.378918000	-0.119962000
F	1.081225000	1.262482000	-2.173135000
C	0.779548000	4.389773000	0.963085000
C	0.228752000	3.136284000	0.442360000
C	-1.124022000	2.810950000	0.575142000
C	-2.068659000	1.705861000	0.336074000
C	-3.436787000	1.876238000	0.003233000
C	-4.019199000	0.594728000	-0.145440000
C	-3.026766000	-0.365985000	0.152718000
C	-2.973230000	-1.830341000	0.178684000
C	-1.693801000	-2.420083000	-0.169324000
C	-1.333461000	-3.699026000	-0.758995000
C	0.019550000	-3.683435000	-0.961312000
C	0.548514000	-2.403656000	-0.523143000
C	1.899459000	-2.064760000	-0.643343000
C	2.818701000	-0.957243000	-0.459987000
C	4.222779000	-1.176018000	-0.264859000
C	4.845828000	0.095769000	-0.163740000
C	3.783114000	1.075911000	-0.298645000
C	3.718485000	2.463808000	-0.020385000
C	2.475870000	3.109869000	0.179937000
C	2.137175000	4.372314000	0.810615000
N	1.319139000	2.455012000	-0.078792000
N	-1.887276000	0.347834000	0.425760000
N	-0.562211000	-1.712323000	-0.021625000
N	2.617745000	0.394612000	-0.510743000
H	-2.018086000	-4.486145000	-1.031770000
H	0.615150000	-4.463238000	-1.417589000
H	-1.681212000	3.665021000	0.945194000
H	2.480692000	-2.934307000	-0.953458000
H	4.622106000	3.025280000	0.170441000
C	-4.111436000	-2.741897000	0.668900000
C	-3.501210000	-3.767048000	1.670399000
C	-5.192085000	-1.968453000	1.459333000
C	-4.789020000	-3.506691000	-0.497310000
H	-2.753659000	-4.417453000	1.213381000
H	-3.031683000	-3.253418000	2.515909000
H	-4.301632000	-4.404529000	2.059405000
H	-5.876262000	-1.414993000	0.820736000
H	-5.801609000	-2.689284000	2.012649000
H	-4.747438000	-1.282341000	2.186494000
H	-5.596527000	-4.127796000	-0.093957000
H	-5.214303000	-2.812831000	-1.222852000
H	-4.091079000	-4.167406000	-1.016839000
H	2.846639000	5.122095000	1.133317000
H	0.182901000	5.162973000	1.428685000
N	4.797252000	-2.456551000	-0.247561000
C	6.062499000	-2.658426000	-0.943246000
H	6.118124000	-3.700290000	-1.279702000
H	6.106696000	-2.008615000	-1.819112000
H	6.947304000	-2.460040000	-0.318561000
C	4.605089000	-3.305447000	0.927116000
H	3.629449000	-3.108729000	1.374637000
H	4.645305000	-4.359597000	0.629576000
H	5.377483000	-3.145613000	1.700004000
N	6.188501000	0.330959000	0.076024000
C	6.806448000	1.572673000	-0.360035000
H	7.867867000	1.391434000	-0.563349000
H	6.333118000	1.924060000	-1.278845000
H	6.746408000	2.365918000	0.403432000
C	6.809690000	-0.254566000	1.261438000
H	6.302088000	-1.175465000	1.539698000

H	7.864003000	-0.475736000	1.063346000
H	6.752803000	0.439452000	2.116158000
N	-4.056220000	3.120469000	-0.362073000
O	-3.699300000	4.144456000	0.242678000
O	-4.891133000	3.103264000	-1.266985000
N	-5.357281000	0.337305000	-0.594954000
O	-5.504877000	-0.544195000	-1.452179000
O	-6.271803000	0.988650000	-0.090768000

E = –2146.4136 a. u.

6. IRC point, BÔB = 189.7°

Ground-state geometry, S_0:

F	-0.266471000	-0.613262000	2.102838000
B	1.341771000	0.977346000	-0.703440000
B	-0.445125000	-0.415322000	0.716123000
O	0.467834000	0.340375000	0.101114000
F	1.089695000	1.060024000	-2.090643000
C	0.851176000	4.366549000	0.810920000
C	0.314373000	3.082615000	0.366491000
C	-1.054014000	2.750554000	0.471995000
C	-1.966988000	1.638172000	0.349939000
C	-3.369452000	1.799320000	0.081557000
C	-3.954540000	0.546796000	-0.041515000
C	-2.949520000	-0.442924000	0.219483000
C	-2.908321000	-1.893649000	0.182927000
C	-1.650155000	-2.485297000	-0.107276000
C	-1.265076000	-3.787723000	-0.624913000
C	0.086899000	-3.763179000	-0.835145000
C	0.598047000	-2.456244000	-0.451528000
C	1.990443000	-2.142612000	-0.503328000
C	2.891294000	-1.053399000	-0.388455000
C	4.336908000	-1.244758000	-0.230983000
C	4.939814000	0.013168000	-0.174527000
C	3.855991000	0.993782000	-0.286614000
C	3.801834000	2.382693000	-0.061291000
C	2.561048000	3.049131000	0.118796000
C	2.215556000	4.342354000	0.676282000
N	1.406155000	2.384923000	-0.099518000
N	-1.805720000	0.273036000	0.443899000
N	-0.493675000	-1.757004000	-0.015977000
N	2.696621000	0.299774000	-0.462262000
H	-1.931390000	-4.599535000	-0.869811000
H	0.690653000	-4.554211000	-1.260107000
H	-1.623324000	3.641293000	0.720749000
H	2.563461000	-3.050681000	-0.684638000
H	4.713120000	2.942121000	0.094753000
C	-4.112725000	-2.809294000	0.520467000
C	-3.624822000	-3.888574000	1.532217000
C	-5.262744000	-2.069035000	1.247762000
C	-4.688079000	-3.494674000	-0.742984000
H	-2.837631000	-4.529312000	1.136560000
H	-3.244495000	-3.416605000	2.444180000
H	-4.469671000	-4.528998000	1.805577000
H	-5.936169000	-1.551003000	0.570124000
H	-5.875698000	-2.807957000	1.771838000
H	-4.890884000	-1.363686000	1.997055000
H	-5.537359000	-4.124896000	-0.456783000
H	-5.036861000	-2.751471000	-1.462130000
H	-3.951877000	-4.130611000	-1.239273000
H	2.918217000	5.112486000	0.963844000
H	0.250558000	5.166031000	1.224479000
N	4.927054000	-2.512603000	-0.197368000
C	6.212179000	-2.688633000	-0.863136000
H	6.318244000	-3.743178000	-1.141624000
H	6.239736000	-2.083209000	-1.770728000
H	7.074665000	-2.412490000	-0.237532000
C	4.745417000	-3.342388000	0.994696000
H	3.767827000	-3.151883000	1.439968000
H	4.803466000	-4.401245000	0.718408000
H	5.515972000	-3.150446000	1.761069000
N	6.290661000	0.267287000	0.044451000
C	6.893185000	1.481839000	-0.483610000
H	7.954221000	1.296784000	-0.687075000
H	6.409026000	1.763342000	-1.420894000
H	6.835412000	2.330460000	0.218822000
C	6.869926000	-0.171999000	1.312287000
H	6.401388000	-1.098862000	1.639509000
H	7.944388000	-0.347439000	1.189160000
H	6.728404000	0.583047000	2.104373000
N	-4.031500000	3.052438000	-0.229464000
O	-3.798171000	4.013165000	0.510243000
O	-4.772574000	3.076516000	-1.208531000
N	-5.296467000	0.342926000	-0.543256000
O	-5.416874000	-0.406423000	-1.513653000
O	-6.213765000	0.924128000	0.030536000

E = –2146.4841 a. u.

Excited-state geometry, S_1:

F	-0.266471000	-0.613262000	2.102838000
B	1.341771000	0.977346000	-0.703440000
B	-0.445125000	-0.415322000	0.716123000
O	0.467834000	0.340375000	0.101114000
F	1.089695000	1.060024000	-2.090643000
C	0.851176000	4.366549000	0.810920000
C	0.314373000	3.082615000	0.366491000
C	-1.054014000	2.750554000	0.471995000
C	-1.966988000	1.638172000	0.349939000
C	-3.369452000	1.799320000	0.081557000
C	-3.954540000	0.546796000	-0.041515000
C	-2.949520000	-0.442924000	0.219483000
C	-2.908321000	-1.893649000	0.182927000
C	-1.650155000	-2.485297000	-0.107276000
C	-1.265076000	-3.787723000	-0.624913000
C	0.086899000	-3.763179000	-0.835145000
C	0.598047000	-2.456244000	-0.451528000
C	1.990443000	-2.142612000	-0.503328000
C	2.891294000	-1.053399000	-0.388455000
C	4.336908000	-1.244758000	-0.230983000
C	4.939814000	0.013168000	-0.174527000
C	3.855991000	0.993782000	-0.286614000
C	3.801834000	2.382693000	-0.061291000
C	2.561048000	3.049131000	0.118796000
C	2.215556000	4.342354000	0.676282000
N	1.406155000	2.384923000	-0.099518000
N	-1.805720000	0.273036000	0.443899000
N	-0.493675000	-1.757004000	-0.015977000
N	2.696621000	0.299774000	-0.462262000
H	-1.931390000	-4.599535000	-0.869811000
H	0.690653000	-4.554211000	-1.260107000
H	-1.623324000	3.641293000	0.720749000
H	2.563461000	-3.050681000	-0.684638000
H	4.713120000	2.942121000	0.094753000

C	-4.112725000	-2.809294000	0.520467000
C	-3.624822000	-3.888574000	1.532217000
C	-5.262744000	-2.069035000	1.247762000
C	-4.688079000	-3.494674000	-0.742984000
H	-2.837631000	-4.529312000	1.136560000
H	-3.244495000	-3.416605000	2.444180000
H	-4.469671000	-4.528998000	1.805577000
H	-5.936169000	-1.551003000	0.570124000
H	-5.875698000	-2.807957000	1.771838000
H	-4.890884000	-1.363686000	1.997055000
H	-5.537359000	-4.124896000	-0.456783000
H	-5.036861000	-2.751471000	-1.462130000
H	-3.951877000	-4.130611000	-1.239273000
H	2.918217000	5.112486000	0.963844000
H	0.250558000	5.166031000	1.224479000
N	4.927054000	-2.512603000	-0.197368000
C	6.212179000	-2.688633000	-0.863136000
H	6.318244000	-3.743178000	-1.141624000
H	6.239736000	-2.083209000	-1.770728000
H	7.074665000	-2.412490000	-0.237532000
C	4.745417000	-3.342388000	0.994696000
H	3.767827000	-3.151883000	1.439968000
H	4.803466000	-4.401245000	0.718408000
H	5.515972000	-3.150446000	1.761069000
N	6.290661000	0.267287000	0.044451000
C	6.893185000	1.481839000	-0.483610000
H	7.954221000	1.296784000	-0.687075000
H	6.409026000	1.763342000	-1.420894000
H	6.835412000	2.330460000	0.218822000
C	6.869926000	-0.171999000	1.312287000
H	6.401388000	-1.098862000	1.639509000
H	7.944388000	-0.347439000	1.189160000
H	6.728404000	0.583047000	2.104373000
N	-4.031500000	3.052438000	-0.229464000
O	-3.798171000	4.013165000	0.510243000
O	-4.772574000	3.076516000	-1.208531000
N	-5.296467000	0.342926000	-0.543256000
O	-5.416874000	-0.406423000	-1.513653000
O	-6.213765000	0.924128000	0.030536000

E = – 2146.3990 a. u.

Excited-state geometry, T_1:

F	-0.452522000	-0.601986000	2.228441000
B	1.237608000	1.030840000	-0.570772000
B	-0.552272000	-0.371636000	0.849378000
O	0.397317000	0.347645000	0.237554000
F	0.883028000	1.090162000	-1.941439000
C	0.784502000	4.426343000	0.953167000
C	0.228291000	3.149061000	0.503091000
C	-1.121332000	2.819464000	0.637146000
C	-2.055801000	1.707597000	0.378007000
C	-3.400224000	1.888993000	-0.034582000
C	-3.988422000	0.613226000	-0.202249000
C	-3.026027000	-0.352584000	0.162242000
C	-2.991240000	-1.817408000	0.184656000
C	-1.706086000	-2.412367000	-0.133238000
C	-1.343341000	-3.674146000	-0.753207000
C	0.013937000	-3.661516000	-0.922332000
C	0.541720000	-2.398852000	-0.433851000
C	1.896198000	-2.071135000	-0.556072000
C	2.825405000	-0.971619000	-0.392679000
C	4.240859000	-1.176943000	-0.283980000
C	4.854922000	0.101419000	-0.208517000
C	3.776747000	1.072463000	-0.273271000
C	3.711178000	2.465557000	-0.019703000
C	2.471418000	3.111327000	0.201224000
C	2.139379000	4.403907000	0.773551000
N	1.311513000	2.441971000	0.008240000
N	-1.894345000	0.346978000	0.498094000
N	-0.575558000	-1.716847000	0.073599000
N	2.609892000	0.376576000	-0.411939000
H	-2.027828000	-4.445267000	-1.068664000
H	0.614556000	-4.427793000	-1.394809000
H	-1.688846000	3.677132000	0.982353000
H	2.464047000	-2.941066000	-0.888740000
H	4.616641000	3.039504000	0.116321000
C	-4.150423000	-2.717671000	0.640053000
C	-3.573457000	-3.739440000	1.664008000
C	-5.248850000	-1.928636000	1.388769000
C	-4.797972000	-3.488424000	-0.539174000
H	-2.818844000	-4.395896000	1.226241000
H	-3.120345000	-3.223401000	2.516833000
H	-4.386843000	-4.370249000	2.036890000
H	-5.896721000	-1.361664000	0.723897000
H	-5.892309000	-2.639895000	1.915499000
H	-4.822077000	-1.250954000	2.134341000
H	-5.624232000	-4.096400000	-0.154185000
H	-5.192139000	-2.799526000	-1.286462000
H	-4.091523000	-4.163687000	-1.027371000
H	2.851588000	5.173945000	1.036946000
H	0.193965000	5.224545000	1.382898000
N	4.829833000	-2.449574000	-0.319565000
C	6.053935000	-2.627515000	-1.091109000
H	6.100052000	-3.663704000	-1.446018000
H	6.039831000	-1.964811000	-1.958235000
H	6.971900000	-2.427993000	-0.516803000
C	4.710230000	-3.321163000	0.847770000
H	3.755973000	-3.146849000	1.347770000
H	4.749733000	-4.369243000	0.529622000
H	5.518616000	-3.163311000	1.583450000
N	6.208970000	0.346094000	-0.049559000
C	6.785030000	1.602011000	-0.501717000
H	7.839201000	1.440879000	-0.753012000
H	6.265741000	1.951515000	-1.396189000
H	6.745050000	2.388796000	0.269947000
C	6.897734000	-0.239401000	1.098537000
H	6.424721000	-1.175435000	1.387294000
H	7.945954000	-0.434189000	0.847924000
H	6.865647000	0.442072000	1.964680000
N	-3.982661000	3.134391000	-0.455918000
O	-3.659138000	4.163213000	0.158852000
O	-4.754912000	3.111922000	-1.414513000
N	-5.301840000	0.361872000	-0.722034000
O	-5.412561000	-0.536666000	-1.566917000
O	-6.234860000	1.034864000	-0.284241000

E = –2146.4157 a. u.

7. IRC point, BÔB = 214.1°

Ground-state geometry, S_0:

F	-0.510485000	-0.679255000	2.117484000
B	1.238659000	1.002197000	-0.636008000
B	-0.502133000	-0.362680000	0.741542000

O	0.546060000	0.410345000	0.399981000
F	0.798743000	0.963648000	-1.977356000
C	0.783322000	4.438887000	0.770949000
C	0.240667000	3.165278000	0.311130000
C	-1.122385000	2.832220000	0.426767000
C	-2.018330000	1.711888000	0.295622000
C	-3.426844000	1.860844000	0.051058000
C	-4.002039000	0.604410000	-0.068236000
C	-2.983070000	-0.374164000	0.167523000
C	-2.941314000	-1.822750000	0.145850000
C	-1.682162000	-2.400001000	-0.158043000
C	-1.295437000	-3.712583000	-0.640075000
C	0.055289000	-3.690579000	-0.852725000
C	0.562677000	-2.374297000	-0.500881000
C	1.953738000	-2.072892000	-0.545471000
C	2.845300000	-0.980293000	-0.444169000
C	4.286186000	-1.162778000	-0.262580000
C	4.878413000	0.098990000	-0.206625000
C	3.787094000	1.070935000	-0.342210000
C	3.725346000	2.455362000	-0.103784000
C	2.482269000	3.116165000	0.063465000
C	2.146327000	4.405608000	0.635457000
N	1.322365000	2.461601000	-0.171284000
N	-1.831658000	0.350131000	0.362553000
N	-0.526936000	-1.652774000	-0.097083000
N	2.635865000	0.369592000	-0.544028000
H	-1.960320000	-4.532737000	-0.859621000
H	0.661770000	-4.490272000	-1.257088000
H	-1.696422000	3.715040000	0.691004000
H	2.526762000	-2.984937000	-0.702742000
H	4.632039000	3.014729000	0.077954000
C	-4.140396000	-2.745861000	0.502118000
C	-3.643326000	-3.817549000	1.517880000
C	-5.293075000	-2.011339000	1.232839000
C	-4.721707000	-3.443049000	-0.752732000
H	-2.853065000	-4.455996000	1.125011000
H	-3.264466000	-3.339021000	2.426870000
H	-4.484372000	-4.461516000	1.794486000
H	-5.978731000	-1.507903000	0.556551000
H	-5.892428000	-2.753013000	1.768528000
H	-4.923369000	-1.295397000	1.973047000
H	-5.564855000	-4.076207000	-0.454988000
H	-5.081093000	-2.706991000	-1.474062000
H	-3.985900000	-4.078168000	-1.250904000
H	2.856701000	5.166226000	0.929210000
H	0.186367000	5.237733000	1.190836000
N	4.881229000	-2.427969000	-0.215278000
C	6.167158000	-2.605954000	-0.877946000
H	6.275758000	-3.661772000	-1.150808000
H	6.195008000	-2.005564000	-1.788958000
H	7.028605000	-2.325205000	-0.252716000
C	4.695935000	-3.252734000	0.979716000
H	3.718676000	-3.057222000	1.423556000
H	4.750860000	-4.312923000	0.707561000
H	5.466448000	-3.060731000	1.746104000
N	6.224538000	0.363717000	0.024725000
C	6.829092000	1.576562000	-0.503541000
H	7.890224000	1.390244000	-0.705886000
H	6.346304000	1.857970000	-1.441544000
H	6.771535000	2.425970000	0.197912000
C	6.804435000	-0.080816000	1.290422000
H	6.333294000	-1.006603000	1.616517000
H	7.878168000	-0.259374000	1.165248000
H	6.666632000	0.673055000	2.084113000
N	-4.103855000	3.107522000	-0.249128000
O	-3.874709000	4.067812000	0.492391000
O	-4.852460000	3.127209000	-1.222690000
N	-5.350028000	0.389428000	-0.554345000
O	-5.474942000	-0.361520000	-1.522859000
O	-6.266037000	0.964610000	0.027228000

E = –2146.4975 a. u.

Excited-state geometry, S_1:

F	-0.837459000	-0.705368000	2.426707000
B	1.220692000	0.930152000	-0.181465000
B	-0.617791000	-0.388649000	1.081830000
O	0.480529000	0.344380000	0.797851000
F	0.774170000	0.710894000	-1.517200000
C	0.693851000	4.521785000	0.614934000
C	0.185994000	3.168842000	0.413310000
C	-1.171624000	2.839939000	0.499794000
C	-2.052322000	1.712828000	0.339199000
C	-3.416379000	1.868059000	-0.094277000
C	-3.976249000	0.588770000	-0.240936000
C	-2.994614000	-0.367375000	0.158703000
C	-2.957914000	-1.814713000	0.199067000
C	-1.682651000	-2.401665000	0.012269000
C	-1.274229000	-3.696863000	-0.495158000
C	0.088360000	-3.673059000	-0.613536000
C	0.576682000	-2.378086000	-0.169937000
C	1.959392000	-2.088109000	-0.206165000
C	2.871865000	-1.007822000	-0.076069000
C	4.328765000	-1.137738000	-0.076023000
C	4.889442000	0.181821000	-0.042388000
C	3.778520000	1.108551000	-0.087820000
C	3.677730000	2.517379000	0.017887000
C	2.434779000	3.144714000	0.154318000
C	2.054982000	4.506885000	0.460447000
N	1.288153000	2.408277000	0.105701000
N	-1.871519000	0.354765000	0.488579000
N	-0.541664000	-1.673800000	0.217135000
N	2.650305000	0.337041000	-0.105465000
H	-1.930434000	-4.495840000	-0.800796000
H	0.716466000	-4.454003000	-1.022298000
H	-1.768830000	3.734093000	0.636102000
H	2.491801000	-2.996723000	-0.462525000
H	4.565881000	3.129196000	0.058860000
C	-4.166599000	-2.723001000	0.511496000
C	-3.707208000	-3.753543000	1.582545000
C	-5.337622000	-1.953346000	1.164785000
C	-4.691164000	-3.474975000	-0.733717000
H	-2.910667000	-4.409367000	1.230073000
H	-3.346456000	-3.240930000	2.480125000
H	-4.559995000	-4.380394000	1.862700000
H	-5.966275000	-1.434669000	0.446276000
H	-5.983353000	-2.674731000	1.674558000
H	-4.984858000	-1.239479000	1.914981000
H	-5.553247000	-4.087586000	-0.446863000
H	-5.006231000	-2.772490000	-1.505340000
H	-3.936818000	-4.141535000	-1.158365000
H	2.737919000	5.338691000	0.567031000
H	0.069726000	5.370442000	0.860044000
N	5.054459000	-2.297804000	-0.118406000
C	6.221393000	-2.439911000	-0.982430000
H	6.021737000	-3.224341000	-1.723638000
H	6.421326000	-1.505455000	-1.503455000

H	7.106809000	-2.733940000	-0.406855000
C	4.641636000	-3.523527000	0.556762000
H	3.859856000	-3.311281000	1.283175000
H	4.289003000	-4.282245000	-0.152877000
H	5.512396000	-3.935587000	1.082115000
N	6.217425000	0.482336000	0.095474000
C	6.744368000	1.780732000	-0.303248000
H	7.785754000	1.649363000	-0.613804000
H	6.178073000	2.171750000	-1.147637000
H	6.724344000	2.503920000	0.523370000
C	7.084441000	-0.259482000	1.008470000
H	6.546852000	-1.097799000	1.446724000
H	7.977068000	-0.629520000	0.491819000
H	7.408330000	0.408816000	1.816845000
N	-4.001970000	3.080878000	-0.572956000
O	-3.693293000	4.148133000	-0.003902000
O	-4.773182000	3.012049000	-1.537625000
N	-5.256218000	0.322987000	-0.820348000
O	-5.311123000	-0.551225000	-1.701067000
O	-6.223807000	0.972034000	-0.414658000

$E = -2146.4513$ a. u.

Excited-state geometry, T_1:

F	-0.822049000	-0.816347000	2.394496000
B	1.183792000	0.936754000	-0.207107000
B	-0.615699000	-0.428643000	1.070519000
O	0.470407000	0.307902000	0.780043000
F	0.611795000	0.820851000	-1.511354000
C	0.741489000	4.488211000	0.870959000
C	0.194328000	3.168872000	0.563440000
C	-1.153313000	2.844179000	0.672284000
C	-2.050985000	1.715152000	0.401150000
C	-3.369733000	1.893280000	-0.090163000
C	-3.938828000	0.617209000	-0.297181000
C	-2.994695000	-0.339751000	0.123379000
C	-2.974106000	-1.802089000	0.150606000
C	-1.680125000	-2.392272000	-0.130924000
C	-1.310274000	-3.642350000	-0.760021000
C	0.052382000	-3.639734000	-0.870836000
C	0.567971000	-2.393077000	-0.332751000
C	1.926819000	-2.087566000	-0.425629000
C	2.843942000	-0.988412000	-0.269234000
C	4.265274000	-1.158770000	-0.283483000
C	4.848316000	0.137677000	-0.240704000
C	3.743335000	1.078529000	-0.196740000
C	3.669011000	2.476735000	0.008217000
C	2.429006000	3.110455000	0.243412000
C	2.094051000	4.454678000	0.674519000
N	1.274665000	2.408093000	0.162322000
N	-1.880313000	0.353675000	0.525623000
N	-0.557608000	-1.701522000	0.141637000
N	2.587198000	0.351087000	-0.224563000
H	-1.991235000	-4.397114000	-1.119262000
H	0.667275000	-4.398118000	-1.337763000
H	-1.743253000	3.713811000	0.940421000
H	2.490154000	-2.955470000	-0.768663000
H	4.566706000	3.075491000	0.055243000
C	-4.142056000	-2.693116000	0.596632000
C	-3.581898000	-3.687352000	1.656188000
C	-5.257437000	-1.885881000	1.298812000
C	-4.763076000	-3.495267000	-0.575703000
H	-2.822290000	-4.354713000	1.242796000
H	-3.137916000	-3.148954000	2.499650000
H	-4.401184000	-4.307812000	2.033756000
H	-5.878139000	-1.322030000	0.605189000
H	-5.923466000	-2.584715000	1.814172000
H	-4.848702000	-1.201339000	2.048179000
H	-5.599716000	-4.090161000	-0.192706000
H	-5.137249000	-2.827624000	-1.351923000
H	-4.047985000	-4.187351000	-1.026320000
H	2.802881000	5.257057000	0.826876000
H	0.146549000	5.328065000	1.204310000
N	4.882930000	-2.410741000	-0.399706000
C	6.030039000	-2.549706000	-1.288229000
H	6.043687000	-3.568703000	-1.692706000
H	5.937037000	-1.848789000	-2.119485000
H	6.996728000	-2.373928000	-0.791124000
C	4.879862000	-3.317817000	0.746131000
H	3.974803000	-3.168956000	1.337234000
H	4.900208000	-4.356077000	0.395488000
H	5.752338000	-3.169450000	1.406588000
N	6.203221000	0.416830000	-0.208460000
C	6.707112000	1.695879000	-0.680273000
H	7.739681000	1.566319000	-1.022326000
H	6.106354000	2.048599000	-1.520804000
H	6.713023000	2.465791000	0.109007000
C	7.024201000	-0.191744000	0.836639000
H	6.592064000	-1.136894000	1.157560000
H	8.037108000	-0.372328000	0.461484000
H	7.089806000	0.469952000	1.715745000
N	-3.932916000	3.134981000	-0.550906000
O	-3.687147000	4.162970000	0.100116000
O	-4.614235000	3.109117000	-1.575871000
N	-5.218121000	0.353779000	-0.887980000
O	-5.282441000	-0.571256000	-1.709241000
O	-6.172898000	1.044438000	-0.530633000

$E = -2146.4327$ a. u.

8. IRC point, BÔB = 233.6°

Ground-state geometry, S_0:

F	-0.741692000	-0.826578000	2.130186000
B	1.175597000	0.930776000	-0.451760000
B	-0.492080000	-0.379892000	0.817244000
O	0.636251000	0.379578000	0.723454000
F	0.494890000	0.725446000	-1.671931000
C	0.751928000	4.472848000	0.673933000
C	0.204078000	3.185427000	0.275488000
C	-1.153597000	2.866942000	0.391037000
C	-2.012195000	1.730489000	0.261956000
C	-3.422558000	1.857115000	0.020373000
C	-3.970352000	0.592397000	-0.102282000
C	-2.929458000	-0.363277000	0.120247000
C	-2.880596000	-1.806584000	0.105934000
C	-1.620991000	-2.370191000	-0.204444000
C	-1.244295000	-3.685374000	-0.679077000
C	0.105497000	-3.673250000	-0.883855000
C	0.614177000	-2.361480000	-0.527802000
C	2.001648000	-2.081813000	-0.572845000
C	2.865595000	-0.979430000	-0.471134000
C	4.306289000	-1.130266000	-0.296477000
C	4.866967000	0.144393000	-0.244232000
C	3.751572000	1.085301000	-0.370310000

C	3.679920000	2.466196000	-0.145067000
C	2.434495000	3.108105000	0.021589000
C	2.112305000	4.423263000	0.530352000
N	1.270468000	2.435047000	-0.164174000
N	-1.782788000	0.375550000	0.316347000
N	-0.464249000	-1.620083000	-0.129416000
N	2.604240000	0.362565000	-0.552185000
H	-1.915769000	-4.499717000	-0.898578000
H	0.712587000	-4.474647000	-1.283672000
H	-1.736973000	3.748290000	0.635672000
H	2.576332000	-2.991974000	-0.730209000
H	4.580718000	3.041034000	0.014494000
C	-4.062378000	-2.740658000	0.491318000
C	-3.531906000	-3.788369000	1.516241000
C	-5.214706000	-2.015319000	1.232201000
C	-4.655214000	-3.463190000	-0.743164000
H	-2.738854000	-4.421826000	1.121145000
H	-3.144278000	-3.287679000	2.409276000
H	-4.358984000	-4.439346000	1.817297000
H	-5.927322000	-1.543347000	0.561238000
H	-5.784122000	-2.758692000	1.797257000
H	-4.845559000	-1.275749000	1.948887000
H	-5.485053000	-4.102256000	-0.421532000
H	-5.036625000	-2.741864000	-1.468102000
H	-3.921818000	-4.096794000	-1.246203000
H	2.832392000	5.193047000	0.771884000
H	0.158739000	5.295441000	1.050854000
N	4.922377000	-2.382960000	-0.228352000
C	6.218136000	-2.552187000	-0.872875000
H	6.341634000	-3.608669000	-1.136859000
H	6.251933000	-1.957818000	-1.787598000
H	7.068061000	-2.258292000	-0.237979000
C	4.731942000	-3.196541000	0.974319000
H	3.752187000	-2.998764000	1.411188000
H	4.791279000	-4.259539000	0.714276000
H	5.498237000	-2.993720000	1.741931000
N	6.206591000	0.436726000	-0.001154000
C	6.800031000	1.658111000	-0.520836000
H	7.865705000	1.486216000	-0.712334000
H	6.323935000	1.934937000	-1.463703000
H	6.724423000	2.505802000	0.181274000
C	6.780446000	0.000089000	1.270775000
H	6.314194000	-0.927949000	1.596840000
H	7.856518000	-0.170486000	1.154286000
H	6.630769000	0.755249000	2.061001000
N	-4.128351000	3.091636000	-0.262828000
O	-3.913449000	4.051500000	0.483747000
O	-4.889238000	3.101988000	-1.227179000
N	-5.323404000	0.352688000	-0.559177000
O	-5.457816000	-0.402095000	-1.523079000
O	-6.235188000	0.912805000	0.043635000

E = –2146.5155 a. u.

Excited-state geometry, S_1:

F	-1.025116000	-0.812946000	2.428768000
B	1.157060000	0.876525000	0.017067000
B	-0.620250000	-0.399556000	1.150579000
O	0.534438000	0.341220000	1.136948000
F	0.573138000	0.559749000	-1.236247000
C	0.652743000	4.534124000	0.575564000
C	0.144517000	3.176161000	0.445265000
C	-1.209914000	2.861600000	0.531372000
C	-2.057705000	1.723922000	0.356887000
C	-3.415564000	1.861874000	-0.101658000
C	-3.939119000	0.575494000	-0.288513000
C	-2.943172000	-0.363171000	0.117176000
C	-2.904918000	-1.803982000	0.159953000
C	-1.624977000	-2.381600000	-0.001908000
C	-1.220018000	-3.684254000	-0.483362000
C	0.144465000	-3.678558000	-0.560076000
C	0.628448000	-2.385876000	-0.114966000
C	2.008588000	-2.108562000	-0.135739000
C	2.889009000	-1.012308000	-0.020581000
C	4.345947000	-1.109354000	-0.063775000
C	4.870618000	0.221224000	-0.062930000
C	3.734107000	1.116957000	-0.070167000
C	3.624095000	2.520564000	0.011277000
C	2.379242000	3.130855000	0.162790000
C	2.009811000	4.506463000	0.402014000
N	1.234574000	2.384188000	0.175475000
N	-1.831698000	0.370929000	0.474502000
N	-0.484467000	-1.652123000	0.227463000
N	2.618075000	0.325089000	-0.031899000
H	-1.878899000	-4.476962000	-0.798192000
H	0.778485000	-4.468344000	-0.941477000
H	-1.814109000	3.752920000	0.651126000
H	2.548282000	-3.018037000	-0.368822000
H	4.503681000	3.145310000	0.009021000
C	-4.111818000	-2.716751000	0.472426000
C	-3.652627000	-3.724302000	1.566088000
C	-5.294927000	-1.947491000	1.104480000
C	-4.622769000	-3.493294000	-0.763052000
H	-2.854477000	-4.386172000	1.229126000
H	-3.294105000	-3.191914000	2.452736000
H	-4.505372000	-4.346408000	1.856567000
H	-5.922623000	-1.444749000	0.374086000
H	-5.938066000	-2.667983000	1.618640000
H	-4.954946000	-1.220712000	1.847978000
H	-5.480147000	-4.109134000	-0.469188000
H	-4.941554000	-2.806118000	-1.546782000
H	-3.861832000	-4.160278000	-1.174726000
H	2.698813000	5.338899000	0.445916000
H	0.029411000	5.394666000	0.776836000
N	5.095340000	-2.253467000	-0.119269000
C	6.246111000	-2.375771000	-1.007263000
H	6.055325000	-3.182893000	-1.725892000
H	6.402099000	-1.447053000	-1.553002000
H	7.154732000	-2.627513000	-0.447810000
C	4.734394000	-3.479710000	0.584437000
H	3.966702000	-3.277707000	1.328589000
H	4.384441000	-4.258271000	-0.104668000
H	5.629555000	-3.859968000	1.092519000
N	6.195370000	0.557014000	0.022653000
C	6.683730000	1.853831000	-0.426435000
H	7.713594000	1.731079000	-0.776811000
H	6.076750000	2.216863000	-1.254746000
H	6.686915000	2.595235000	0.384091000
C	7.107682000	-0.146114000	0.921942000
H	6.605534000	-0.990848000	1.388944000
H	7.994831000	-0.500483000	0.385345000
H	7.436300000	0.544041000	1.709922000
N	-4.009576000	3.072337000	-0.573508000
O	-3.745539000	4.132856000	0.031838000
O	-4.745033000	3.010193000	-1.566730000
N	-5.191698000	0.287523000	-0.910636000
O	-5.202599000	-0.593966000	-1.787655000

O	-6.184092000	0.923323000	-0.543150000

$E = -2146.4678$ a. u.

Excited-state geometry, T_1:

F	-1.034299000	-0.935926000	2.351533000
B	1.129916000	0.872632000	-0.015813000
B	-0.628130000	-0.445067000	1.104540000
O	0.521303000	0.288907000	1.100554000
F	0.450259000	0.652638000	-1.248256000
C	0.683422000	4.510478000	0.792596000
C	0.144911000	3.175085000	0.565945000
C	-1.203250000	2.865477000	0.650832000
C	-2.066602000	1.725486000	0.384840000
C	-3.386637000	1.882031000	-0.110708000
C	-3.916743000	0.597607000	-0.351555000
C	-2.949540000	-0.338451000	0.059452000
C	-2.921653000	-1.797220000	0.096828000
C	-1.622199000	-2.374023000	-0.178426000
C	-1.253319000	-3.630483000	-0.787185000
C	0.111264000	-3.644039000	-0.859928000
C	0.619494000	-2.399031000	-0.317451000
C	1.978159000	-2.108962000	-0.374573000
C	2.862015000	-0.995752000	-0.212357000
C	4.284917000	-1.128366000	-0.271768000
C	4.829272000	0.183124000	-0.249840000
C	3.699831000	1.088628000	-0.162294000
C	3.612775000	2.484880000	0.015920000
C	2.369301000	3.101235000	0.251226000
C	2.034673000	4.467381000	0.595878000
N	1.220702000	2.380193000	0.227098000
N	-1.847290000	0.367790000	0.474643000
N	-0.503887000	-1.678647000	0.111410000
N	2.557955000	0.334324000	-0.141742000
H	-1.935868000	-4.379998000	-1.153916000
H	0.733891000	-4.411579000	-1.300784000
H	-1.803386000	3.741708000	0.868988000
H	2.552809000	-2.975825000	-0.698287000
H	4.500149000	3.099909000	0.021369000
C	-4.070600000	-2.690513000	0.586941000
C	-3.477405000	-3.649004000	1.661729000
C	-5.182259000	-1.878267000	1.289061000
C	-4.704034000	-3.530078000	-0.551890000
H	-2.725757000	-4.325090000	1.247773000
H	-3.012999000	-3.081603000	2.474098000
H	-4.283438000	-4.261685000	2.078566000
H	-5.818462000	-1.333608000	0.593871000
H	-5.834069000	-2.572660000	1.828042000
H	-4.767713000	-1.176103000	2.018564000
H	-5.526215000	-4.123608000	-0.136913000
H	-5.100656000	-2.887674000	-1.338306000
H	-3.990084000	-4.226882000	-0.997194000
H	2.743812000	5.278810000	0.685816000
H	0.082569000	5.367895000	1.064868000
N	4.933716000	-2.360415000	-0.412745000
C	6.055278000	-2.467877000	-1.337398000
H	6.077108000	-3.482730000	-1.751869000
H	5.923341000	-1.761474000	-2.158598000
H	7.032716000	-2.277399000	-0.867567000
C	4.988494000	-3.273035000	0.727571000
H	4.101474000	-3.146483000	1.350288000
H	5.019970000	-4.309184000	0.371509000
H	5.879813000	-3.107056000	1.357720000
N	6.175843000	0.501378000	-0.270752000
C	6.632565000	1.785011000	-0.776547000
H	7.652007000	1.671666000	-1.161373000
H	5.989877000	2.116346000	-1.594379000
H	6.657148000	2.563895000	0.003396000
C	7.053477000	-0.081055000	0.742641000
H	6.656564000	-1.033090000	1.088360000
H	8.053771000	-0.241785000	0.326732000
H	7.140577000	0.588835000	1.613491000
N	-3.976434000	3.119151000	-0.551024000
O	-3.787304000	4.133216000	0.139827000
O	-4.622203000	3.103476000	-1.598875000
N	-5.183391000	0.305173000	-0.951373000
O	-5.225137000	-0.636719000	-1.755873000
O	-6.152367000	0.990366000	-0.621037000

$E = -2146.4462$ a. u.

9. Akamptisomer $\boldsymbol{t_2}$, BÔB = 240.8°

Ground-state geometry, S_0:

F	-1.091088000	-0.850891000	2.445953000
B	1.107192000	0.870255000	0.106168000
B	-0.613647000	-0.387299000	1.208983000
O	0.527173000	0.371893000	1.290748000
F	0.418373000	0.565338000	-1.099151000
C	0.633304000	4.566525000	0.525315000
C	0.117175000	3.209425000	0.441257000
C	-1.238027000	2.892858000	0.496558000
C	-2.056219000	1.731996000	0.356022000
C	-3.403295000	1.836901000	-0.125044000
C	-3.911902000	0.563962000	-0.296621000
C	-2.920013000	-0.365488000	0.144554000
C	-2.877913000	-1.805252000	0.179627000
C	-1.597727000	-2.381966000	0.046166000
C	-1.193206000	-3.700226000	-0.387109000
C	0.169508000	-3.700594000	-0.452319000
C	0.649256000	-2.390997000	-0.053562000
C	2.038118000	-2.110130000	-0.105045000
C	2.867632000	-0.987369000	-0.119819000
C	4.330608000	-1.069171000	-0.243745000
C	4.822134000	0.228302000	-0.192605000
C	3.665448000	1.105180000	-0.053628000
C	3.590686000	2.496464000	0.019323000
C	2.343844000	3.127547000	0.178390000
C	1.990793000	4.517026000	0.363234000
N	1.199741000	2.394850000	0.222532000
N	-1.825799000	0.383217000	0.511075000
N	-0.452220000	-1.640813000	0.258902000
N	2.540929000	0.335016000	-0.026082000
H	-1.850520000	-4.503071000	-0.678703000
H	0.807755000	-4.502615000	-0.798455000
H	-1.848696000	3.785178000	0.581044000
H	2.622016000	-3.017554000	-0.235986000
H	4.497974000	3.084739000	-0.031406000
C	-4.101553000	-2.711201000	0.472677000
C	-4.573834000	-3.508702000	-0.766180000
C	-3.680630000	-3.693383000	1.607345000
C	-5.310423000	-1.929699000	1.044452000
H	-4.882628000	-2.836653000	-1.568028000
H	-3.798296000	-4.170632000	-1.156264000
H	-5.430950000	-4.130577000	-0.485201000

H	-3.341916000	-3.140105000	2.488986000
H	-4.545486000	-4.301856000	1.890943000
H	-2.879216000	-4.371049000	1.313036000
H	-5.975965000	-2.638839000	1.544900000
H	-5.004358000	-1.187775000	1.787951000
H	-5.913529000	-1.445112000	0.280763000
N	4.995425000	-2.301997000	-0.300046000
N	6.114784000	0.738910000	-0.326412000
C	6.072786000	-2.560605000	0.645789000
H	5.864645000	-2.040107000	1.583253000
H	6.116986000	-3.636723000	0.853484000
H	7.065686000	-2.250018000	0.285675000
C	5.125634000	-2.958273000	-1.596672000
H	5.995233000	-2.597429000	-2.171564000
H	5.239803000	-4.038923000	-1.452849000
H	4.226123000	-2.781836000	-2.191484000
C	7.022025000	0.117596000	-1.281335000
H	7.585612000	-0.732715000	-0.869383000
H	6.457117000	-0.224014000	-2.151010000
H	7.748412000	0.867091000	-1.616210000
C	6.760547000	1.271877000	0.871841000
H	6.024594000	1.777689000	1.500077000
H	7.236921000	0.478341000	1.472127000
H	7.531157000	1.995473000	0.584671000
H	2.690591000	5.341256000	0.374308000
H	0.019072000	5.442428000	0.686454000
N	-5.147693000	0.287228000	-0.992348000
O	-5.101862000	-0.546019000	-1.898881000
O	-6.150185000	0.902789000	-0.636665000
N	-4.041525000	3.056724000	-0.585439000
O	-4.558568000	3.041444000	-1.699246000
O	-4.012239000	4.030273000	0.173222000

E = –2146.5215 a. u.

Excited-state geometry, S_1:

F	-1.089820000	-0.846469000	2.402129000
B	1.134831000	0.852835000	0.084632000
B	-0.628063000	-0.397955000	1.151304000
O	0.551788000	0.316194000	1.238265000
F	0.509383000	0.552839000	-1.147906000
C	0.676783000	4.521734000	0.675090000
C	0.151515000	3.174160000	0.519408000
C	-1.205721000	2.880342000	0.586954000
C	-2.052194000	1.751888000	0.369022000
C	-3.399646000	1.899463000	-0.112644000
C	-3.921836000	0.615749000	-0.322370000
C	-2.934851000	-0.327084000	0.093433000
C	-2.916999000	-1.766638000	0.154315000
C	-1.646218000	-2.363408000	0.004630000
C	-1.258778000	-3.689235000	-0.418601000
C	0.106885000	-3.707038000	-0.485147000
C	0.603692000	-2.400480000	-0.105216000
C	1.985374000	-2.134615000	-0.167208000
C	2.866540000	-1.038970000	-0.161090000
C	4.325920000	-1.134960000	-0.232961000
C	4.849294000	0.192335000	-0.171420000
C	3.718985000	1.081624000	-0.009904000
C	3.623157000	2.480531000	0.106640000
C	2.383183000	3.098962000	0.257461000
C	2.033841000	4.476492000	0.510390000
N	1.230243000	2.365668000	0.246456000
N	-1.822801000	0.399250000	0.469358000
N	-0.495862000	-1.636310000	0.204288000
N	2.597998000	0.296326000	-0.033760000
H	-1.927543000	-4.485071000	-0.703184000
H	0.731471000	-4.521464000	-0.828440000
H	-1.803469000	3.774247000	0.718634000
H	2.510719000	-3.066771000	-0.313248000
H	4.506592000	3.099196000	0.105955000
C	-4.136244000	-2.653320000	0.492655000
C	-4.655355000	-3.460185000	-0.719456000
C	-3.689255000	-3.631080000	1.618445000
C	-5.311136000	-1.852048000	1.099202000
H	-4.973865000	-2.791776000	-1.519543000
H	-3.899294000	-4.141303000	-1.116877000
H	-5.514384000	-4.063837000	-0.405841000
H	-3.317555000	-3.074939000	2.484652000
H	-4.551378000	-4.228254000	1.932511000
H	-2.903749000	-4.317285000	1.301408000
H	-5.968016000	-2.551481000	1.624871000
H	-4.964633000	-1.114519000	1.828919000
H	-5.926764000	-1.356050000	0.354022000
N	5.098920000	-2.265645000	-0.274598000
N	6.166621000	0.534920000	-0.312369000
C	6.264020000	-2.420707000	0.592319000
H	6.428771000	-1.513983000	1.170909000
H	6.083520000	-3.254022000	1.283460000
H	7.163033000	-2.650179000	0.008824000
C	4.725700000	-3.473761000	-0.999662000
H	5.627857000	-3.877178000	-1.474554000
H	4.323179000	-4.248002000	-0.333998000
H	4.000402000	-3.240008000	-1.777107000
C	7.028544000	-0.111994000	-1.298876000
H	7.931500000	-0.516344000	-0.827789000
H	6.494074000	-0.914947000	-1.802088000
H	7.335303000	0.630479000	-2.046685000
C	6.695901000	1.787210000	0.211138000
H	6.153809000	2.077707000	1.109886000
H	7.747047000	1.631775000	0.473981000
H	6.646994000	2.596269000	-0.530064000
H	2.736073000	5.296837000	0.568965000
H	0.062754000	5.387927000	0.880506000
N	-5.160667000	0.329314000	-0.968287000
O	-5.161211000	-0.573048000	-1.825555000
O	-6.154908000	0.984854000	-0.640460000
N	-3.975939000	3.118784000	-0.582386000
O	-4.684769000	3.074859000	-1.595562000
O	-3.721743000	4.169189000	0.045090000

E = –2146.4698 a. u.

Excited-state geometry, T_1:

F	-1.149428000	-0.894130000	2.363574000
B	1.089501000	0.868611000	0.089284000
B	-0.656573000	-0.406395000	1.142669000
O	0.491573000	0.338801000	1.249536000
F	0.425936000	0.590904000	-1.134414000
C	0.606623000	4.555759000	0.652490000
C	0.086724000	3.205269000	0.493446000
C	-1.263668000	2.894143000	0.529100000
C	-2.101059000	1.734477000	0.301856000
C	-3.412463000	1.848414000	-0.226000000
C	-3.911252000	0.547571000	-0.436965000
C	-2.935960000	-0.352706000	0.029405000
C	-2.895816000	-1.808604000	0.145682000

C	-1.582872000	-2.386074000	-0.093726000
C	-1.199644000	-3.671741000	-0.619268000
C	0.167976000	-3.689757000	-0.646272000
C	0.658150000	-2.410829000	-0.176976000
C	2.012108000	-2.108593000	-0.260915000
C	2.867382000	-0.969690000	-0.189678000
C	4.305085000	-1.057564000	-0.303307000
C	4.806449000	0.249765000	-0.178055000
C	3.657516000	1.110061000	-0.017043000
C	3.570153000	2.506253000	0.105005000
C	2.317649000	3.129114000	0.264710000
C	1.964705000	4.510677000	0.507695000
N	1.175739000	2.394352000	0.252042000
N	-1.855352000	0.382727000	0.441139000
N	-0.477763000	-1.665041000	0.179954000
N	2.529469000	0.338601000	-0.028320000
H	-1.873070000	-4.442668000	-0.957397000
H	0.803865000	-4.481842000	-1.019462000
H	-1.877689000	3.778229000	0.661488000
H	2.605900000	-2.990343000	-0.494268000
H	4.475181000	3.100528000	0.087411000
C	-4.024736000	-2.675432000	0.718036000
C	-4.658547000	-3.604487000	-0.349404000
C	-3.407764000	-3.543640000	1.853841000
C	-5.138596000	-1.824276000	1.367589000
H	-5.086448000	-3.026175000	-1.168701000
H	-3.935524000	-4.312093000	-0.761918000
H	-5.456293000	-4.188673000	0.122522000
H	-2.935436000	-2.911638000	2.611618000
H	-4.203174000	-4.128356000	2.327398000
H	-2.656653000	-4.244433000	1.481506000

H	-5.790456000	-2.485129000	1.947191000
H	-4.724966000	-1.078673000	2.053232000
H	-5.773712000	-1.323300000	0.638846000
N	4.965415000	-2.298188000	-0.409424000
N	6.107271000	0.755903000	-0.221975000
C	5.927568000	-2.639004000	0.631621000
H	5.541427000	-2.315649000	1.601509000
H	6.054326000	-3.727479000	0.659238000
H	6.923182000	-2.189289000	0.486042000
C	5.280269000	-2.798879000	-1.744074000
H	6.215962000	-2.388288000	-2.157647000
H	5.379999000	-3.889966000	-1.708840000
H	4.465849000	-2.549926000	-2.429298000
C	7.062528000	0.165626000	-1.148254000
H	7.544989000	-0.746817000	-0.766041000
H	6.561550000	-0.068489000	-2.089522000
H	7.850642000	0.898666000	-1.353172000
C	6.692213000	1.217361000	1.037774000
H	5.927395000	1.694961000	1.652889000
H	7.129659000	0.386302000	1.616339000
H	7.482229000	1.947258000	0.831492000
H	2.666607000	5.330997000	0.566525000
H	-0.009563000	5.424232000	0.843203000
N	-5.159279000	0.201487000	-1.044853000
O	-5.174679000	-0.801549000	-1.773305000
O	-6.139915000	0.904378000	-0.795706000
N	-4.015906000	3.062314000	-0.715803000
O	-4.608559000	3.010282000	-1.792430000
O	-3.887230000	4.090791000	-0.033546000

$E = -2146.4431$ a. u.

6. References

(1) Frisch, M. J.; Trucks, G. W.; Schlegel, H. B.; Scuseria, G. E.; Robb, M. A.; Cheeseman, J. R.; Scalmani, G.; Barone, V.; Petersson, G. A.; Nakatsuji, H.; Li, X.; Caricato, X.; Marenich, A.; Bloino, J.; Janesko, B. G.; Gomperts, R.; Mennucci, B.; Hratchian, H. P.; Ortiz, J. V; Izmaylov, A. F.; Sonnenberg, J. L.; Williams-Young, D.; Ding, F.; Lipparini, F.; Egidi, F.; Goings, J.; Peng, B.; Petrone, A.; Henderson, T.; Ranasinghe, D.; Zakrzewski, V. G.; Gao, J.; Rega, N.; Zheng, G.; Liang, W.; Hada, M.; Ehara, M.; Toyota, K.; Fukuda, R.; Hasegawa, J.; Ishida, M.; Nakajima, T.; Honda, Y.; Kitao, O.; Nakai, H.; Vreven, T.; Throssell, K.; Montgomery, J. A.; Peralta, J. E.; Ogliaro, F.; Bearpark, M.; Heyd, J. J.; Brothers, E.; Kudin, K. N.; Staroverov, V. N.; Keith, T.; Kobayashi, R.; Normand, J.; Raghavachari, K.; Rendell, A.; Burant, J. C.; Iyengar, S. S.; Tomasi, J.; Cossi, M.; Millam, J. M.; Klene, M.; Adama, C.; Cammi, R.; Ochterski, J. W.; Martin, R. L.; Morokuma, K.; Farkas, O.; Foresman, J. B.; Fox, D. J. Gaussian 09, Revision D.01. Gaussian Inc: Wallingford, CT 2016.

(2) Becke, A. D. Density-functional Thermochemistry. III. The Role of Exact Exchange. *J. Chem. Phys.* **1993**, *98* (7), 5648–5652. https://doi.org/10.1063/1.464913.

(3) Becke, A. D. A New Mixing of Hartree–Fock and Local Density-Functional Theories. *J. Chem. Phys.* **1993**, *98* (2), 1372–1377. https://doi.org/10.1063/1.464304.

(4) Ditchfield, R.; Hehre, W. J.; Pople, J. A. Self-Consistent Molecular-Orbital Methods. IX. An Extended Gaussian-Type Basis for Molecular-Orbital Studies of Organic Molecules. *J. Chem. Phys.* **1971**, *54* (2), 724–728. https://doi.org/10.1063/1.1674902.

(5) Gordon, M. S.; Binkley, J. S.; Pople, J. A.; Pietro, W. J.; Hehre, W. J. Self-Consistent Molecular-Orbital Methods. 22. Small Split-Valence Basis Sets for Second-Row Elements. *J. Am. Chem. Soc.* **1982**, *104* (10), 2797–2803. https://doi.org/10.1021/ja00374a017.

(6) Grimme, S.; Antony, J.; Ehrlich, S.; Krieg, H. A Consistent and Accurate Ab Initio Parametrization of Density Functional Dispersion Correction (DFT-D) for the 94 Elements H-Pu. *J. Chem. Phys.* **2010**, *132* (15), 154104. https://doi.org/10.1063/1.3382344.

(7) de Andrade, K. N.; Raffaeli, N. M.; Fiorot, R. G. Akamptisomerism as a Switching Element: Substituent Effects on Bond Angle Reflection and Photophysical Properties of B–O–B Porphyrins. *Inorg. Chem.* **2025**, *64* (23), 11683–11695. https://doi.org/10.1021/acs.inorgchem.5c01132.

(8) de Andrade, K.; Rocha, P.; de Miranda, D.; Raffaeli, N.; Ferreira, G.; Fiorot, R. Akamptisomerism Beyond Porphyrins: Bond Angle Reflection and Stereochemical Divergences in Corrole- and Porphyrin-Anchored BOB Bridges. *Inorg. Chem.* **2026**.

(9) Canfield, P. J.; Blake, I. M.; Cai, Z.-L.; Luck, I. J.; Krausz, E.; Kobayashi, R.; Reimers, J. R.; Crossley, M. J. A New Fundamental Type of Conformational Isomerism. *Nat. Chem.* **2018**, *10* (6), 615–624. https://doi.org/10.1038/s41557-018-0043-6.

(10) Yanai, T.; Tew, D. P.; Handy, N. C. A New Hybrid Exchange–Correlation Functional Using the Coulomb-Attenuating Method (CAM-B3LYP). *Chem. Phys. Lett.* **2004**, *393* (1–3), 51–57. https://doi.org/10.1016/j.cplett.2004.06.011.

(11) Drzewiecka-Matuszek, A.; Rutkowska-Zbik, D. Application of TD-DFT Theory to Studying Porphyrinoid-Based Photosensitizers for Photodynamic Therapy: A Review. *Molecules* **2021**, *26* (23), 7176. https://doi.org/10.3390/molecules26237176.

(12) Mack, J.; Stone, J.; Nyokong, T. Trends in the TD-DFT Calculations of Porphyrin and Phthalocyanine Analogs. *J. Porphyr. Phthalocyanines* **2014**, *18* (08n09), 630–641. https://doi.org/10.1142/S108842461450045X.

(13) Cai, Z.-L.; Crossley, M. J.; Reimers, J. R.; Kobayashi, R.; Amos, R. D. Density Functional Theory for Charge Transfer: The Nature of the N-Bands of Porphyrins and Chlorophylls Revealed through CAM-B3LYP, CASPT2, and SAC-CI Calculations. *J. Phys. Chem. B* **2006**, *110* (31), 15624–15632. https://doi.org/10.1021/jp063376t.

(14) Rätsep, M.; Cai, Z.-L.; Reimers, J. R.; Freiberg, A. Demonstration and Interpretation of Significant Asymmetry in the Low-Resolution and High-Resolution *Q y* Fluorescence and Absorption Spectra of Bacteriochlorophyll *a*. *J. Chem. Phys.* **2011**, *134* (2). https://doi.org/10.1063/1.3518685.

(15) Carneiro, L. M.; Keppler, A. F.; Ferreira, F. F.; Homem-de-Mello, P.; Bartoloni, F. H. Mechanisms for the Deactivation of the Electronic Excited States of α-(2-Hydroxyphenyl)- *N* -Phenylnitrone: From Intramolecular Proton and Charge Transfer to Structure Twisting and Aggregation. *J. Phys. Chem. B* **2022**, *126* (38), 7373–7384. https://doi.org/10.1021/acs.jpcb.2c03924.

(16) Jesus, A. J. L.; Lucena, J. R.; Rodrigues, G. P.; Ildiz, G. O.; do Monte, S. A.; Ventura, E.; Fausto, R. Infrared Spectrum and <scp>UV</Scp> -Triggered Transformations of Matrix-Isolated *Meta* -Fluorothiophenol Supported by Ground and Excited State Theoretical Calculations. *J. Comput. Chem.* **2025**, *46* (6). https://doi.org/10.1002/jcc.70045.

(17) de Souza, J. R.; de Moraes, M. M. F.; Aoto, Y. A.; Homem-de-Mello, P. Can One Use the Electronic Absorption Spectra of Metalloporphyrins to Benchmark Electronic Structure Methods? A Case Study on the Cobalt Porphyrin. *Physical Chemistry Chemical Physics* **2020**, *22* (41), 23886–23898. https://doi.org/10.1039/D0CP04699J.

(18) de Souza, B.; Farias, G.; Neese, F.; Izsák, R. Predicting Phosphorescence Rates of Light Organic Molecules Using Time-Dependent Density Functional Theory and the Path Integral Approach to

Dynamics. *J. Chem. Theory Comput.* **2019**, *15* (3), 1896–1904. https://doi.org/10.1021/acs.jctc.8b00841.

(19) Neese, F.; Wennmohs, F.; Becker, U.; Riplinger, C. The ORCA Quantum Chemistry Program Package. *J. Chem. Phys.* **2020**, *152* (22). https://doi.org/10.1063/5.0004608.

(20) Neese, F. Software Update: The <scp>ORCA</Scp> Program System—Version 5.0. *WIREs Computational Molecular Science* **2022**, *12* (5). https://doi.org/10.1002/wcms.1606.

(21) Plasser, F. TheoDORE: A Toolbox for a Detailed and Automated Analysis of Electronic Excited State Computations. *J. Chem. Phys.* **2020**, *152* (8). https://doi.org/10.1063/1.5143076.

(22) El-Sayed, M. A. Triplet State. Its Radiative and Nonradiative Properties. *Acc. Chem. Res.* **1968**, *1* (1), 8–16. https://doi.org/10.1021/ar50001a002.

(23) Guo, H.; Zheng, S.; Chen, S.; Han, C.; Yang, F. A First Porphyrin Liquid Crystal with Strong Fluorescence in Both Solution and Aggregated States Based on the AIE-FRET Effect. *Soft Matter* **2019**, *15* (41), 8329–8337. https://doi.org/10.1039/C9SM01174A.

(24) Xu, M.; Li, X.; Liu, S.; Zhang, L.; Xie, W. Near-Infrared Organic Light-Emitting Materials, Devices and Applications. *Mater. Chem. Front.* **2023**, *7* (20), 4744–4767. https://doi.org/10.1039/D3QM00585B.

(25) Teixeira, R.; Serra, V. V.; Botequim, D.; Paulo, P. M. R.; Andrade, S. M.; Costa, S. M. B. Fluorescence Spectroscopy of Porphyrins and Phthalocyanines: Some Insights into Supramolecular Self-Assembly, Microencapsulation, and Imaging Microscopy. *Molecules* **2021**, *26* (14), 4264. https://doi.org/10.3390/molecules26144264.
